# Mapping Evaluation Use: A Scoping Review of Extant Literature (2005–2022)

**DOI:** 10.1177/10982140241234841

**Published:** 2024-03-13

**Authors:** Michelle Searle, Amanda Cooper, Paisley Worthington, Jennifer Hughes, Rebecca Gokiert, Cheryl Poth

**Affiliations:** 1 4257 Queen's University, Kingston, Ontario, Canada; 2 3158 University of Alberta, Edmonton, Alberta, Canada

**Keywords:** evaluation use, research synthesis, evaluation practice, theory-practice relationship

## Abstract

Factors influencing evaluation use has been a primary concern for evaluators. However, little is known about the current conceptualizations of evaluation use including what counts as use, what efforts encourage use, and how to measure use. This article identifies enablers and constraints to evaluation use based on a scoping review of literature published since 2009 (*n* = 47). A fulsome examination to map factors influencing evaluation use identified in extant literature informs further study and captures its evolution over time. Five factors were identified that influence evaluation use: (1) resources; (2) stakeholder characteristics; (3) evaluation characteristics; (4) social and political environment; and (5) evaluators characteristics. Also examined is a synthesis of practical and theoretical implications as well as implications for future research. Importantly, our work builds upon two previous and impactful scoping reviews to provide a contemporary assessment of the factors influencing evaluation use and inform consequential evaluator practice.

Evaluations and their use are essential for generating evidence about the efficacy and effectiveness of programs and policies. Evaluations typically involve data-informed processes that culminate in results that are shared in formal and informal ways to improve programs and decision-making (Alkin & King, 2016; Preskill & Torres, 2000; Weiss, 1998). As Coffman and Beer (2019) commented, “evaluators are constantly trying to deliver value amidst change.” When thinking about the value of evaluation, use is a key consideration in efforts to optimize evaluation processes, make data relevant to end-users, and increase the relevancy of recommendations and the uptake of findings that may improve programs. Peck and Gorzalski (2009) state that, “it is clear the field of program evaluation needs to continue to research into the area of evaluation use” (p. 152). Evaluation use is recognized as a dynamic concept that has long been a topic of focus in evaluation scholarship and practice (Christie, 2007). The importance of the Cousins and Leithwood (1986) and Johnson et al. (2009) reviews about evaluation use and utilization to the field is clear: they are highly cited (706 and 478 citations reported, respectively, on Google Scholar, as of December 15, 2023). This article explores evaluation use since 2005 to provide a comprehensive examination of evaluation use in the scholarly literature.

Evaluation use and utilization are not defined consistently and are often used interchangeably. Alkin and King (2017) explain, “the use continuum extends from nonuse to use and reflects the extent to which someone does something with an evaluation, although measuring the extent of use may present a nontrivial challenge” (p. 436). The Oxford Dictionary (2023) defines use as “putting something to work… for any (esp. a beneficial or productive) purpose” while utilize and utilization are explained as making something useful or the action of utilizing. We continue to use these terms interchangeably as both use and utilize relate to the application of evaluation thinking enacted through evaluation processes, products, or findings, which enable those involved to make use of new information. This article focuses on evaluation use and builds on two previous reviews of evaluation use considering use patterns from 1971 to 1985 (Cousins & Leithwood, 1986) and from 1986 to 2005 (Johnson et al., 2009).

Terminology is enmeshed in the dialogue about evaluation use (King & Alkin, 2019). Use, utilization, and influence are some of the terms attributed to the concept of evaluation use; while some authors define the term(s) they choose, many do not (Johnson et al., 2009; Mark, 2011; Peterson & Skolits, 2020). In this article, we focus on evaluation use as it relates to evaluation participants engaging in the evaluation process, incorporating findings into program improvement, and contributing to decision-making. Although various concepts of evaluation use emerged decades ago (Cousins & Leithwood, 1986), discussions of what counts as use, how to encourage use, and how to measure use continue to spark discussion among evaluation theorists, scholars, and practitioners (King & Alkin, 2019; Patton, 2020b).

Over the last three decades, evaluation has shifted from traditional goals, such as producing reports for funders and decision-makers, toward more involved and iterative processes (e.g., collaborative approaches to evaluation [Shulha et al., 2016], developmental evaluation [Patton, 2011], and interactive evaluation [Stevahn & King, 2016]). These approaches (and others) focus on stakeholder engagement^1^ by examining how evaluations can be used in real time to innovate and adapt programs or initiatives as they unfold in complex social settings (Fleischer & Christie, 2009; Preskill & Caracelli, 1997). Over time, the discipline of evaluation has made its deep commitment to use and utilization more explicit by highlighting its presence in professional evaluation standards (Yarbrough et al., 2010), evaluation approaches (Patton, 1997; Stufflebeam & Zhang, 2017), and theories of stakeholder engagement (Boyce & Chouinard, 2017; Cousins, 2020; Hood et al., 2015; O'Sullivan, 2012; Shulha et al., 2016). As the field of evaluation has continued to evolve, so has the construct of evaluation use, establishing an ongoing need for research.

In this article, we describe the results of our scoping review, which builds on previous reviews (Cousins & Leithwood, 1986; Johnson et al., 2009) and synthesizes the relevant international evidence on evaluation use that has emerged over the past 17 years (2005–2022). The initial use review undertaken by Cousins and Leithwood (1986) focused on “two major factors shown to influence evaluation use, (1) the characteristics of the evaluation implementation, and (2) characteristics of the decision or policy setting” (p. 332). Their literature search found 65 empirical studies that identify factors that influence use, including evaluation quality, evaluator credibility, relevance, and communication quality. In the next major review of evaluation use, Johnson and colleagues (2009) examined 41 studies that revealed recent theoretical articles pushing forward conversations about evaluation use that go beyond focusing on the main types (e.g., process/findings) toward envisioning a nuanced reconceptualization that includes intangible and indirect forms of use. They noted that two categories and 12 characteristics from Cousins and Leithwood (1986) still hold, with an even distribution between evaluation implementation versus decision-making and policy-setting. Shulha and Cousins (1997) added evaluator competence as a characteristic and stakeholder involvement as a new category. They also point out that use literature has expanded to focus on the centrality of contexts, the growth of use beyond individuals to include organizations, and the expanding roles of evaluators (Shulha & Cousins, 1997). Our review flagged process use as a recent concept, albeit without a substantive body of literature, as the evaluation field remains more focused on outcomes and results. Since the concept of use continues to evolve, there is a need to aggregate our understanding of the evaluation-use literature to better inform practice and make sense of evaluation use in our complex, diverse, and evolving post-pandemic sociopolitical landscape.

This article extends the recent work of evaluation scholars who compiled theoretical issues regarding evaluation use (Alkin & King, 2016; Mark, 2011; Patton, 2020b) and examined the quality of empirical works on evaluation use (Brandon & Singh, 2009). The objectives of our review are to (1) examine the range and nature of the empirical studies and literature on evaluation use published over the past 17 years, and (2) examine these studies to identify factors and findings that influence evaluation use.

## Method

**Figure 1.**
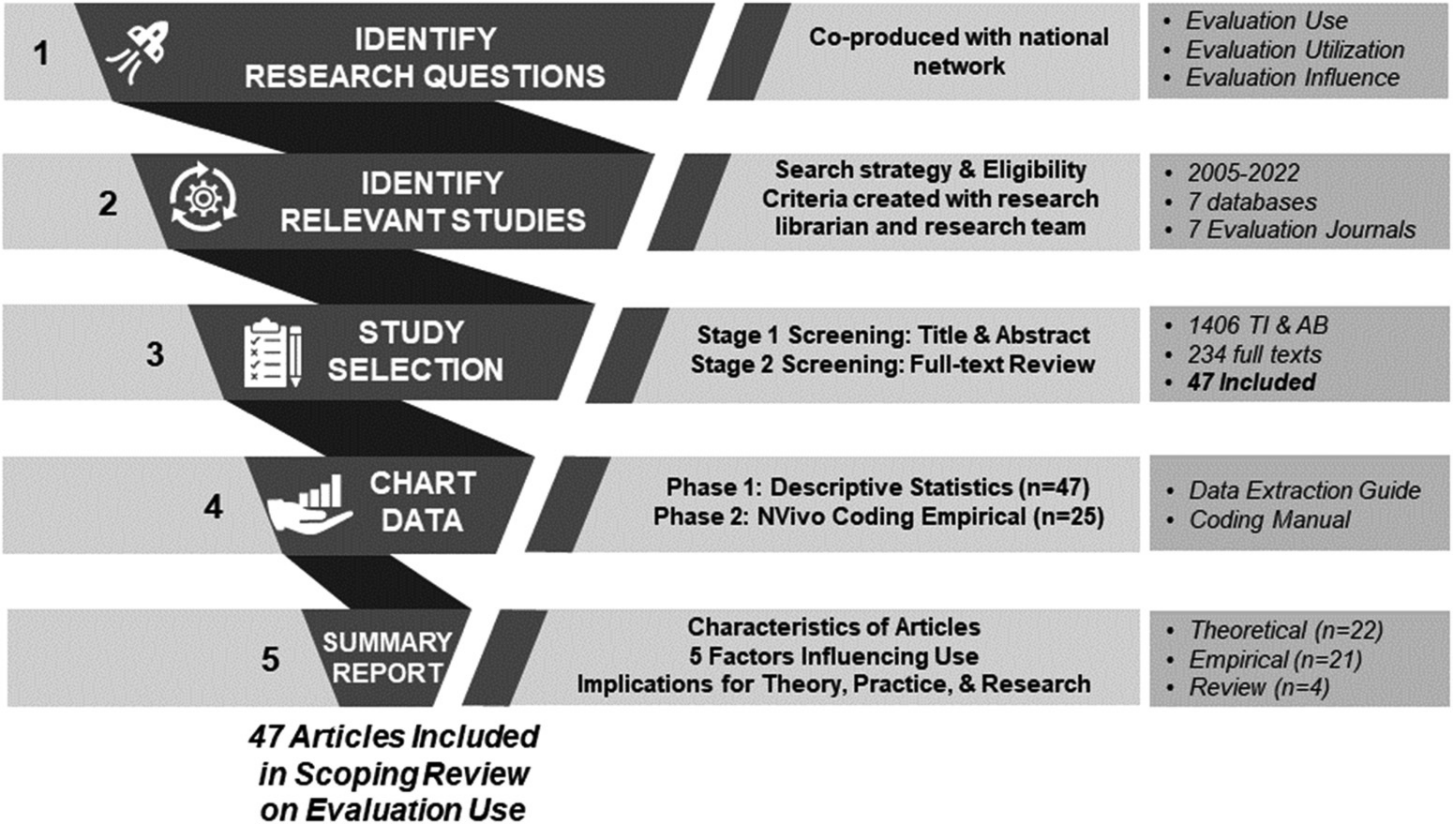
Procedural description of the five stages of our scoping review.

To guide our scoping review, we used the methodological framework developed by Arksey and O’Malley (2005), which includes identifying relevant sources of evidence, selecting sources, charting the data, and summarizing results. A procedural description of our scoping review can be found in Figure 1, which we developed using the Preferred Reporting Items for Systematic reviews and Meta-Analyses extension for Scoping Reviews (PRISMA-ScR). Each aspect of Figure 1 is explained in the subsequent sections.

We began this review in 2021 but were slowed by the pandemic; therefore, the authors conducted a more recent search in June 2022. The goal of this review is meta-aggregative, meaning we sought not to reinterpret the examined articles but to describe the sample by generating descriptive statistics, reliably summarizing the key factors found to influence evaluation use, and coalescing the reported implications for theory, practice, and future research, as originally outlined by the authors included in the review.

### Identifying Research Questions

This article is one of five scoping reviews undertaken by an interdisciplinary and intersectoral Evaluation Capacity Network (ECN; https://www.evaluationcapacitynetwork.com/) that is funded by a national social science funder to understand existing knowledge and practices of evaluation within the social sector, including (1) evaluation use and influence, (2) evaluation capacity building, (3) culturally responsive evaluation, (4) community-driven evaluation, and (5) evaluation in early childhood development. As part of the relationship with the ECN, the authors and other members of the network generated the search parameters and criteria jointly to establish guideposts for the authors of these reviews.

Our review was guided by this question: What factors emerge from the current extant literature on evaluation use that can positively influence use with diverse stakeholders and inform implications for theory, research, and practice in the field of evaluation? To operationalize this broad question and guide this review, the authors developed four research questions:

1. What are the characteristics of the extant literature on evaluation use (geographical origin, types of articles, contexts, methods used in empirical work, sectors/disciplines)?
2. What terminology and definitions are being used for evaluation use?
3. What empirical studies exist and what factors arise from that work that influence the use of evaluation findings in different contexts?
4. What implications emerge from the extant literature in the following three areas: theoretical implications, practical implications for evaluators, and implications for future research on evaluation use across diverse stakeholders?

### Identifying Relevant Studies

The ECN and a research librarian worked with the authors to develop a protocol to guide study selection, eligibility criteria, data extraction, and data analysis. Criteria included keywords, publication period, language, study designs, and settings (Table 1). The initial searches conducted by the lead author and research assistants (Searle, Hughes & Worthington) yielded 2,318 titles and abstracts based on these eligibility requirements.

**Table 1.** Search Parameters for the Scoping Review.

Criteria	Details
Keywords	evaluation use, evaluation utilization, evaluation influence
Publication years	2005–2022
Language	English
Types of articles	Journal articles, theoretical articles, empirical articles Exclusions: grey literature, theses
Databases	Academic Search Complete Education Source PsycINFO Canadian Business & Current Affairs (CBCA) Education Resources Information Center (ERIC) Education Database
Key journals	*American Journal of Evaluation* *Canadian Journal of Program Evaluation* *New Directions for Evaluation* *Journal of Multidisciplinary Evaluation* *Evaluation* *Evaluation and Program Planning* *Evaluation Journal of Australasia* *Evaluation in the Health Professions*

### Selecting the Studies

Working with a research librarian, three researchers (Reed, Searle, Hughes & Worthington) screened the studies using Covidence (Covidence, 2020), a web-based application that streamlines reviews (Harrison et al., 2020). The researchers used a two-step process, first reviewing the title and abstracts of each retained article and then conducting a full-text review of each. Disagreements were resolved by a fourth screener (Cooper). The research team met at the start of the extraction process and again before initiating the full-text article review to ensure the scope of the review was feasible. Records that were not articles or did not address our objectives were excluded. The researchers then analyzed the final sample size of 47 articles.

### Charting the Data

Using the extracted data, the authors created categories using a cloud-based spreadsheet (Airtable, 2022) to chart article characteristics (year, geographic location, type of article, sector/discipline), the study context (purpose, terms used, definitions, methods, participants), and article contributions (findings, theoretical implications, practice implications, future research). The first four authors piloted a small sample of articles (*n* = 5) to check for agreement. Two research assistants and two experienced researchers (Hughes, Worthington, Searle & Cooper) spot-checked the extracted data for consistency and accuracy.

### Summarizing and Reporting

The researchers divided the 47 articles into three categories for further analysis: empirical articles (*n* = 21), theoretical articles (*n* = 22), and reviews (scoping/systematic review) (*n* = 4). Results were synthesized in two phases based on type of article: phase 1 generated descriptive statistics and frequencies related to relevant categories; phase 2 narrowed the sample to empirical articles (*n* = 21) for further analysis and coding using NVivo software (QSR International Pty Ltd., 2020) to summarize the factors influencing evaluation use.

## Findings

**Figure 2.**
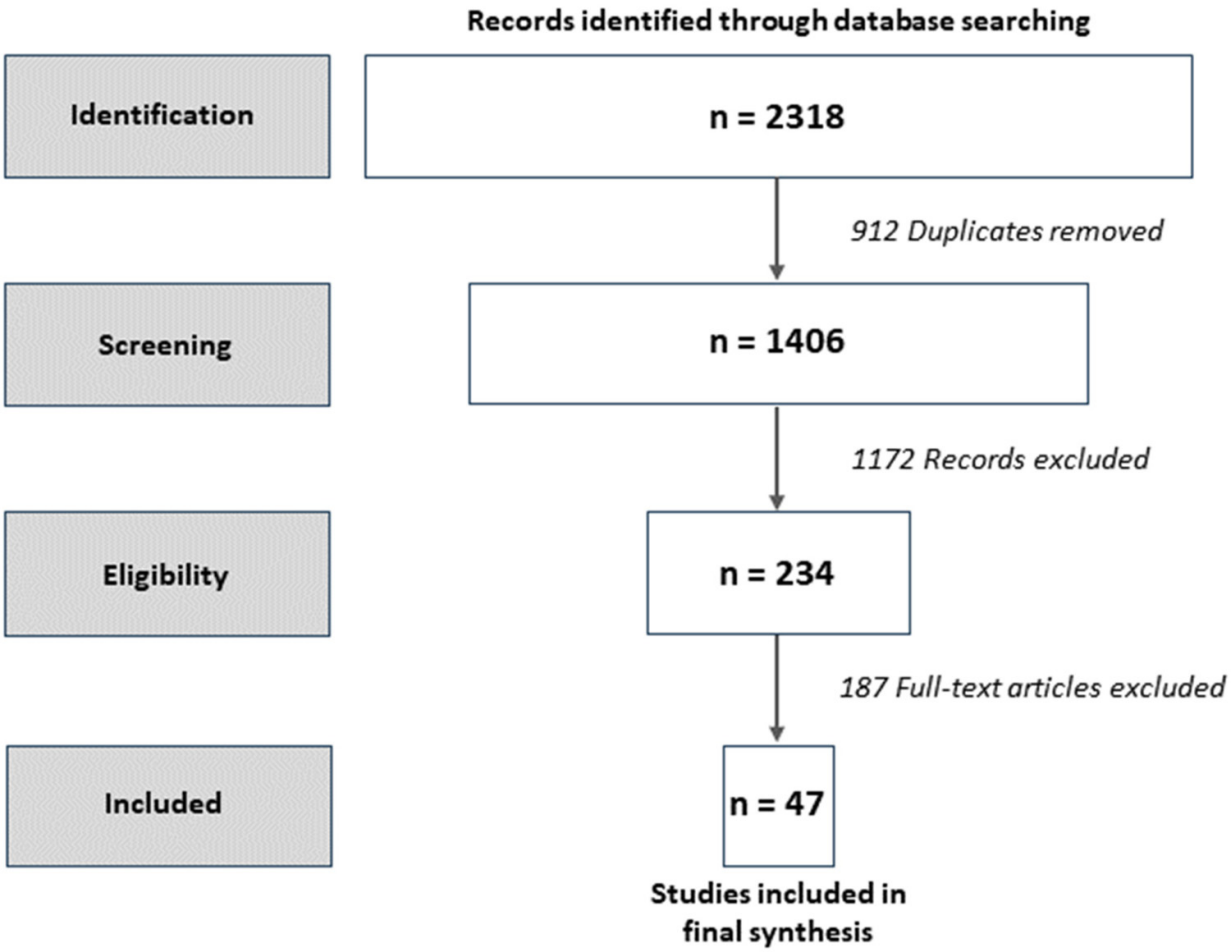
PRISMA diagram of scoping review identification, screening, eligibility, and inclusion.

The search of databases and evaluation journals returned 2,318 articles. The first screening resulted in 234 full-text articles, which were assessed for eligibility. The second screening identified 47 articles for a full-text review, as reported in Figure 2 using the PRISMA-ScR (Tricco et al., 2018). Appendix A provides summaries of the 47 articles organized into theoretical and empirical categories. This review yielded important findings about the terminology and definitions as well as key characteristics of the evaluation-use and utilization literature published between 2005 and 2022. We identified five factors about evaluation use and utilization and examined implications from the empirical studies. Our focus on the empirical studies was purposeful for two reasons: (1) to align with previous use reviews (Cousins & Leithwood, 1986; Johnson et al., 2009) and (2) to respect publishing (word count) while exploring the integration of theory and practice within the complex situations where we are cultivating knowledge about evaluation use and utilization.

### Terminology and Definitions of Evaluation Use

From the 47 articles reviewed, 28 unique terms emerged to describe evaluation use (Table 2), while one article discussed evaluation use without employing a use term (Cook, 2014).

**Table 2.** Prevalence of Terms Used to Conceptualize Evaluation Use.

Term used in articles (*n* = 47)	Occurrences (%)	Defined	Not defined
Use	29 (61.2%)	11	18
Influence	10 (21.3%)	8	2
Utilization	10 (21.3%)	3	7
Conceptual/enlightenment use	6 (12.8%)	6	0
Direct/instrumental use	6 (12.8%)	6	0
Process use	6 (12.8%)	5	1
Political/symbolic use	3 (6.4%)	3	0
Findings use	3 (6.4%)	1	2
Nonuse/misuse/nonmisuse	2 (4.3%)	2	0
Utility	2 (4.3%)	2	0
Active/passive	1 (2.1%)	1	0
Evaluation champion	1 (2.1%)	1	0
Evaluation literacy	1 (2.1%)	1	0
Imposed + central + local use^1^	1 (2.1%)	1	0
Legitimizing + tactical use^1^	1 (2.1%)	1	0
Knowledge translation	1 (2.1%)	0	1
Impact of evaluation	1 (2.1%)	0	1
Information use	1 (2.1%)	0	1
Usefulness	1 (2.1%)	0	1
No term identified	1 (2.1%)	–	–

1 Some articles used a unique cluster of terminology not found in other articles.

2 Percentages will not add to 100 as each term created unique total and percentage of analysis was based on that specific total rather than across the full data set.

Across the sample, 17 articles (36.2%) provided definitions for their chosen term(s). Although the 47 articles employed various terms, 29 articles (68.1%) employed the term “use” (see Table 3 for sample definitions of most frequently cited terms). Of the articles that employed the term use, 18 (62.1%) did not provide a definition, suggesting the term may serve as catch-all language to encompass numerous aspects related to evaluation use. This observation supports previous literature suggesting a lack of clarity and consistency in use of terminology (Alkin & King, 2016, 2017; King & Alkin, 2019).

**Table 3.** Sample Definitions for the Three Most Common “Use” Terms in Articles.

Term	Definition	Citation
Use	“We employ the term ‘use’ rather than ‘influence’ although we use ‘use’ broadly. We attempt to identify it as ‘process use’ or ‘use of findings’ and classify it as instrumental, conceptual, or symbolic.”	Johnson et al. (2009), p. 86
	“By this time, we have come to reasonable, but not universal, agreement on a definition of use, that is, the effect the evaluation has on the evaluand—the ‘thing’ being evaluated—and those connected to the evaluand.”	Christie, (2007), p. 8
Influence	“[Influence is] the capacity or power of persons or things to produce effects on others by intangible or indirect means.”	Kirkhart, (2000), p. 7	
	“Evaluation influence explicitly includes both changes that take place at the location and general time frame of the evaluation and changes that take place elsewhere and later. Influence also accommodates those cases in which change takes place because of an evaluation, but individuals involved are unaware of the role of evaluation in that change.”	Mark, (2011), p. 113
Utilization	“Utilization-focused (UFE) evaluation is a pragmatic model of evaluation based on the principle that evaluations should be done ‘for and with specific intended primary users for specific, intended uses’ (Patton, 1997, p. 37).”	Peterson & Skolits, (2020), p. 3

Two-thirds of articles (*n* = 31, 66.0%) adopted one single term to describe use, with the remaining one-third (*n* = 16, 34.0%) adopting numerous terms to position their conceptualization of evaluation use. Of the articles using numerous terms, 13 (81.3%) defined terms and compared definitions within the article.

### Characteristics of Evaluation Utilization Literature

A full set of characteristics of the articles included in this review are represented in Appendix B with key details to follow. The number of studies (*n* = 47) may be considered small, yet it is important to note the ever-increasing total number of evaluation-use publications since 2005. The peak publication period was between 2014 with 10 articles (21.3%) and 2019 with 14 articles (29.8%). Based on the affiliated country of each first author, 70.2% of the sample was rooted in North America (United States, *n* = 26; Canada, *n* = 7). This dominance may be the result of the search parameters used to identify articles from eight major evaluation journals, many of which are based in North America. Various sectors were represented, with 61.7% of the articles written in the context of evaluation as an independent field with interdisciplinary applications (*n* = 29). Methodology was also explored and is discussed in the empirical studies section.

### Theoretical Studies

Keeping in mind our overall goal of generating a broad understanding of the field of evaluation-use literature in the last 17 years (2005–2022), we noted that theoretical articles featured a wide variety of topics related to evaluation use. Specifically, these articles explored terminology (Amo & Cousins, 2008; Mark, 2011; Patton, 2007), the history of both knowledge use (Blake & Ottoson, 2009; Donnelly & Searle, 2017) and evaluation use (Alkin & King, 2016; Patton, 2020b), program evaluation standards (Yarbrough, 2017), similarities and differences among dimensions of use in different contexts (Appleton-Dyer et al., 2012; Brandon, 2011; Lawrenz et al., 2011), evaluation use frameworks (Conner et al., 2012; Peterson & Skolits, 2020), strategies to increase use (Cook, 2014; Liket et al., 2014), reflections on failed evaluations (Sturges, 2015), the integration of evaluation use and technology (Svensson & Cousins, 2015), the relevance of evaluation use for addressing equity and social disparity (Mark, 2017), evaluation capacity building through game-based learning (Olejniczak, 2017; Olejniczak et al., 2020), the impact of evaluation use (Granger & Maynard, 2015), and identification of the roles of practice organizations (King & Alkin, 2019). The theoretical studies informed our reading of the empirical studies.

### Empirical Studies

Of the empirical articles, qualitative methodologies (*n* = 9) were most prominent (Adams et al., 2015; Larsson, 2021; Ledermann, 2012; Marra, 2021; Marshall et al., 2022; Milzow et al., 2019; Ramírez et al., 2017; Rogers et al., 2019; Whitmore et al., 2017), followed by quantitative (*n* = 7) studies (Azzam, 2011; Baughman et al., 2012; Christie, 2007; Clinton, 2014; Froncek & Rohmann, 2019; Mason & Azzam, 2019; Vanlandingham, 2011), with five studies using mixed methods (Bourgeois & Naré, 2015; Breidahl et al., 2017; Donnelly et al., 2014; Fleischer & Christie, 2009; Lawrenz et al., 2007). Most of the studies used a case study design and did not explicitly include impact metrics.

We excluded review articles (*n* = 4) from the analysis of stakeholders sampled in the empirical studies (*n* = 21). Fifteen of the empirical studies sampled one stakeholder group (71%). However, because six studies sampled multiple stakeholder groups, the total number of stakeholders mentioned (27) exceeds the number of empirical articles (*n* = 21). The types of stakeholders mentioned included evaluators (26%), government/policy-makers (26%), and service delivery staff (26%), whereas leaders/managers were mentioned in three articles (11%), community stakeholders in 2 (7%), and the public in 1 (4%).

### Factors Influencing Use

The research reported here provides a review of the factors that were present in the empirical literature published between 2005 and 2022. Although previous research has examined the methodological rigor of research on evaluation (e.g., Brandon & Singh, 2009), the purpose of this study was to synthesize ideas on evaluation use. Importantly, we note that many of the identified studies seemed to operate under the assumption that increased use is beneficial. While there are plausible scenarios in which evaluators use evaluations for less-than-positive ends, the concept of inappropriate use was rarely discussed in the empirical articles.

**Figure 3.**
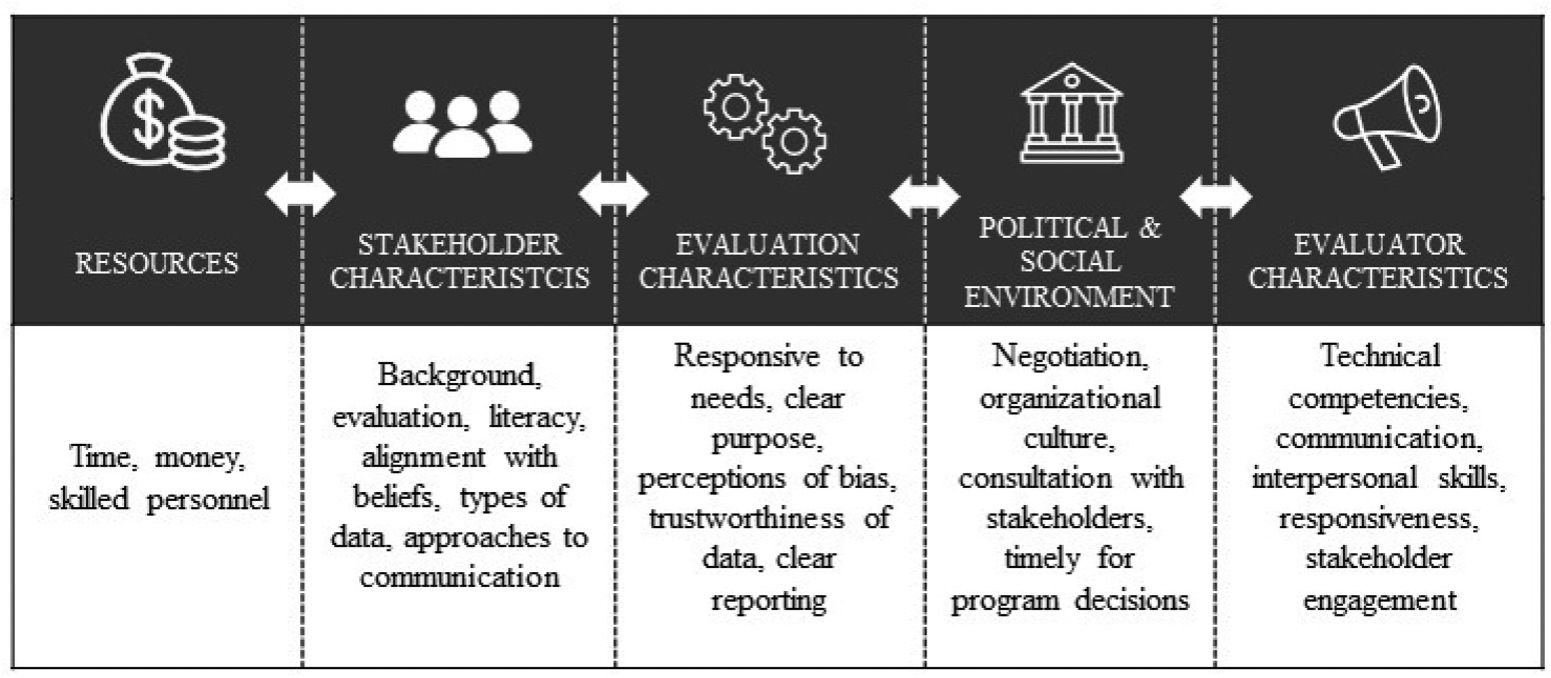
Five categories of factors influencing evaluation use.

The empirical studies discussed numerous factors that influence evaluation use. While these factors are presented separately, there is considerable overlap and influence on each. For example, the role of stakeholders’ features throughout each of these factors in terms of how that role relates to use, but it is also a standalone factor. Most of these factors are related to the evaluation context and had the power to either enable or constrain evaluation use. Five categories of factors emerged: resources, stakeholder characteristics, evaluation characteristics, social and political environment, and evaluator characteristics (see Figure 3). Findings for each of these factors are discussed subsequently.

### Resources for Evaluations

Limited resources are an oft-cited challenge affecting evaluation inquiries and their use. This factor emerged in three articles (Adams et al., 2015; Donnelly & Searle, 2017; Milzow et al., 2019), underlining the importance of having sufficient time and funds to adequately prepare for use activities throughout and following the evaluation inquiry. In addition, the availability of skilled personnel (Donnelly & Searle, 2017) for both conducting a useful evaluation and supporting its use after the inquiry were identified as critical factors. The impact was reportedly greater when the necessary resources were available (although this was usually a qualitative description not linked to explicit impact metrics). The availability of resources is mentioned first, as it is a foundational factor that played a role in all other factors.

### Stakeholder Characteristics

Numerous articles mentioned the stakeholder qualities that impacted the level of evaluation use. Use was affected, for example, when stakeholders already had opinions about the program and evaluation (Christie, 2007; Ledermann, 2012; Mason & Azzam, 2019). When challenging stakeholders’ preformed opinions, evaluations had to be of high quality (Ledermann, 2012; Mason & Azzam, 2019). The professional backgrounds of primary stakeholders strongly predicted how they would respond to evaluation results (Christie, 2007), depending on their preferences in terms of the types of data collected and the approaches used to communicate findings (Mason & Azzam, 2019). The types of evaluation data that stakeholders desired and took seriously varied with discipline (Christie, 2007). Use increased when there was alignment between the evaluation data and the preferences of stakeholders and decreased when there was misalignment. In cases of misalignment, some stakeholders were rejecting the data and making the decisions that disregarded the evaluation evidence (Fleischer & Christie, 2009; Mason & Azzam, 2019; Whitmore et al., 2017).

Increased evaluation literacy among evaluation users (Rogers et al., 2019) heightens openness to the evaluation data and, consequently, supports evaluation use. Some studies found that involving stakeholders in all steps of the evaluation had a positive impact on stakeholder opinion and support their buy-in while also increasing evaluation capacity (Fleischer & Christie, 2009). Establishing evaluation experiences as well as positive social connections with stakeholders and those who are in the stakeholders’ networks garnered excitement and enthusiasm for the practice of evaluation and using the results (Ramírez et al., 2017; Rogers et al., 2019).

### Evaluation Characteristics

The characteristics of the evaluation were a recurring thread across our sample. Evaluation management affected evaluation use (Ledermann, 2012; Whitmore et al., 2017) with use reportedly higher when evaluation management responds to the various needs of stakeholders (Rogers et al., 2019). Reiterating the evaluation's purpose and situating it within the organization's goals have a positive impact on use, particularly when positioning the evaluation as an opportunity for continued learning (Ramírez et al., 2017; Rogers et al., 2019; Whitmore et al., 2017). Evaluations with clearly defined and managed evaluator-stakeholder relationships (Ledermann, 2012; Whitmore et al., 2017) and clear leadership among the stakeholders resulted in higher use (Whitmore et al., 2017). Further, healthy team building where stakeholders are involved in various aspects of the evaluation and invited to critically reflect on it also heightens evaluation use (Clinton, 2014; Whitmore et al., 2017).

Evaluation quality and methodological choice matter (Christie, 2007; Ledermann, 2012). Evaluations without a clear purpose (Bourgeois & Naré, 2015; Rogers et al., 2019; Whitmore et al., 2017), novel findings (Ledermann, 2012), and actual or perceived low quality (Ledermann, 2012; Mason & Azzam, 2019) did not yield high use. Evaluations with novel findings but that yield what the stakeholders consider to be high-quality results tend to facilitate use; however, these characteristics are not consistently required for facilitating use (Ledermann, 2012). Interestingly, while low-quality evaluations could potentially trigger conflict within the program and evaluation, they were considered acceptable by stakeholders when they reaffirmed stakeholders’ preformed opinions (Ledermann, 2012). The question of quality becomes more important when challenging stakeholders’ existing opinions (Ledermann, 2012; Mason & Azzam, 2019); balancing broad data with in-depth information according to stakeholder preferences can enhance use (Christie, 2007).

Other factors reducing evaluation use included stakeholders’ perceptions of bias (Fleischer & Christie, 2009; Ledermann, 2012), substance (Ledermann, 2012; Mason & Azzam, 2019), and trustworthiness (Mason & Azzam, 2019; Rogers et al., 2019). Clear evaluation reporting and design are important factors when aiming to facilitate use; modern data-visualization techniques can weaken some users’ perceptions of the evaluation's rigor and thoroughness (Bourgeois & Naré, 2015; Mason & Azzam, 2019). Evaluators need to include sufficient detail in reports to allow readers to make their own interpretations of the findings (Mason & Azzam, 2019).

### Political and Social Environment

Some articles suggest that the social and political contexts of the evaluand are more important to facilitating use than core evaluation activities (Christie, 2007; Fleischer & Christie, 2009; Ledermann, 2012; Milzow et al., 2019; Ramírez et al., 2017; Rogers & Gullickson, 2018; Vanlandingham, 2011; Whitmore et al., 2017). Lower use was reported when stakeholders perceived the evaluation as an opportunity to negotiate aspects of the program rather than an analytical process that challenged them to use data to inform discussions (Breidahl et al., 2017). Unless consulted, stakeholders responsible for implementing postevaluation program changes did not perceive the evaluation findings as helpful (Breidahl et al., 2017). In some circumstances, the evaluation was commissioned after program decisions were already made, resulting in nonuse (Ledermann, 2012). While social and political factors can lead to a lack of evaluation use (Breidahl et al., 2017; Fleischer & Christie, 2009; Vanlandingham, 2011), evaluators who remain sensitive to these factors can encourage use (Appleton-Dyer et al., 2012; Breidahl et al., 2017; Rogers et al., 2021). Organizational cultures that embrace learning were more likely to use evaluation findings (Ramírez et al., 2017; Whitmore et al., 2017), especially in the presence of an evaluation champion (Rogers & Gullickson, 2018).

### Evaluator Characteristics

Evaluators themselves influence the level of use. Underlining every evaluation are the evaluator's interpersonal skills (Rogers et al., 2019; Whitmore et al., 2017), technical competencies (Ledermann, 2012), and communication (Bourgeois & Naré, 2015; Rogers et al., 2019; Whitmore et al., 2017). Beyond these, evaluator traits fall primarily into two categories: responsiveness to stakeholder needs (Azzam, 2011; Rogers et al., 2019; Whitmore et al., 2017) and commitment to stakeholder engagement (Azzam, 2011; Clinton, 2014; Fleischer & Christie, 2009; Whitmore et al., 2017).

As discussed above, stakeholders have varying needs related to evaluation literacy (Rogers et al., 2019), data literacy (Bourgeois & Naré, 2015), preferences (Christie, 2007), and evaluation capacity (Clinton, 2014; Ramírez et al., 2017; Rogers et al., 2019; Rogers & Gullickson, 2018). Evaluators who recognize the needs of stakeholders and actively address them through evaluation design, activities, or other efforts are more likely to have these stakeholders use the evaluation findings (Azzam, 2011; Bourgeois & Naré, 2015; Clinton, 2014; Whitmore et al., 2017). For example, stakeholders sometimes require additional support to fully digest evaluation reports (Bourgeois & Naré, 2015; Rogers et al., 2019); evaluators who overlook this need are less likely to have findings used.

Use appears to be highest when trusting partnerships exist (Ledermann, 2012). These partnerships include evaluators who value stakeholder input (Azzam, 2011) and stakeholders who are committed to participating in the evaluation (Ramírez et al., 2017; Rogers & Gullickson, 2018; Whitmore et al., 2017). Evaluations with power imbalances between stakeholders and evaluators were less likely to result in use and had higher attrition rates; stakeholder perceptions of fairness had a large impact on whether findings were used and whether individuals continued to contribute (Froncek & Rohmann, 2019). Pseudo-participatory activities, which seek feedback from stakeholders but do not ensure the feedback is incorporated, result in less use than nonparticipatory evaluations with no stakeholder involvement (Froncek & Rohmann, 2019). Evaluators wishing to involve stakeholders must intend to use stakeholder feedback; otherwise, they will jeopardize both their relationships and the use of the inquiry (Froncek & Rohmann, 2019; Whitmore et al., 2017).

Evaluators who view themselves more as facilitators than experts and who believe in the importance of stakeholder engagement tend to see more evaluation use (Azzam, 2011; Fleischer & Christie, 2009; Milzow et al., 2019; Rogers et al., 2019). These evaluators aim to break down social barriers while using their interpersonal skills and humor to address evaluation anxiety (Rogers et al., 2019). Internal evaluators are also positioned to establish a sense of cohesion and mutual understanding with stakeholders by leveraging social and professional relationships (Fleischer & Christie, 2009; Rogers et al., 2019). Evaluators seeking public recognition and media coverage decreased use, especially in legislative contexts (Vanlandingham, 2011).

### Implications

Many articles included in our review shared implications, which we have synthesized into three broad areas: practical implications, theoretical implications, and suggestions for future research. Each of these areas is discussed to point toward opportunities for thinking about evaluation use and utilization.

#### Practical Implications

Sixteen articles (Adams et al., 2015; Azzam, 2011; Bourgeois & Naré, 2015; Breidahl et al., 2017; Christie, 2007; Clinton, 2014; Donnelly & Searle, 2017; Fleischer & Christie, 2009; Froncek & Rohmann, 2019; Lawrenz et al., 2011; Ledermann, 2012; Mason & Azzam, 2019; Milzow et al., 2019; Ramírez et al., 2017; Vanlandingham, 2011; Whitmore et al., 2017) share practical implications for evaluators. Many recommendations focus on better understanding the evaluation audience by building community with stakeholders, sharing power, and maintaining open communication (Breidahl et al., 2017; Christie, 2007; Donnelly & Searle, 2017; Froncek & Rohmann, 2019; Ledermann, 2012; Mason & Azzam, 2019; Ramírez et al., 2017; Vanlandingham, 2011; Whitmore et al., 2017). The articles recommend continued critical reflection, professional development, and networking for evaluators to increase their competence in approaches that may appeal to different stakeholders (Azzam, 2011; Breidahl et al., 2017; Fleischer & Christie, 2009; Ledermann, 2012; Mason & Azzam, 2019; Milzow et al., 2019).

Another practical suggestion focused on incorporating active sessions with stakeholders to increase evaluation literacy and the collaborative interpretation of findings (Adams et al., 2015; Breidahl et al., 2017; Christie, 2007; Donnelly & Searle, 2017; Lawrenz et al., 2011; Ramírez et al., 2017; Whitmore et al., 2017). Designing an evaluation with its use in mind from the beginning is important in facilitating that use, as is conducting use-planning sessions throughout the inquiry (Adams et al., 2015; Breidahl et al., 2017; Clinton, 2014). Including actionable and relevant recommendations in reports (Adams et al., 2015; Bourgeois & Naré, 2015; Lawrenz et al., 2011; Mason & Azzam, 2019; Vanlandingham, 2011) that consider both stakeholders’ data literacy and the effect of recommendations (Froncek & Rohmann, 2019) further facilitate use by helping stakeholders identify next steps. Given the importance of social and political contexts in evaluation use, many articles recommended that evaluators remain perceptive of and sensitive to these environmental factors and conscious of how they can influence the evaluation (Adams et al., 2015; Breidahl et al., 2017; Christie, 2007; Donnelly & Searle, 2017; Whitmore et al., 2017).

#### Theoretical Implications

Thirteen articles shared theoretical implications (Azzam, 2011; Brandon & Singh, 2009; Breidahl et al., 2017; Christie, 2007; Donnelly & Searle, 2017; Fleischer & Christie, 2009; Froncek & Rohmann, 2019; Ledermann, 2012; Mason & Azzam, 2019; Ramírez et al., 2017; Rogers et al., 2019; Vanlandingham, 2011; Whitmore et al., 2017). In one study (Brandon & Singh, 2009), the researchers note there is an apparent lack of methodological soundness within the empirical literature on evaluation use. Further, they note that most studies examine the relationship between evaluation factors and evaluation use rather than the levels and mechanisms of use (Brandon & Singh, 2009).

In most articles, the researchers report the importance of familiarizing oneself with the program background, evaluation context, and social complexities surrounding the inquiry (Donnelly & Searle, 2017; Ledermann, 2012; Ramírez et al., 2017; Whitmore et al., 2017). External and internal evaluators play different roles in facilitating use, with internal evaluators leveraging established social connections and external evaluators assuming additional roles such as negotiator, facilitator, and listener (Fleischer & Christie, 2009; Rogers et al., 2019). Beyond the evaluators, research identified that stakeholders’ perceptions of data and evaluation have a strong bearing on evaluation design, engagement techniques, and reporting styles (Azzam, 2011; Breidahl et al., 2017; Froncek & Rohmann, 2019; Mason & Azzam, 2019; Whitmore et al., 2017). The articles observe a breadth of factors that influence use, ranging from interpersonal considerations to internally held opinions and perceptions to sense-making sessions. Evaluators who adopt a wider definition of use—such as the concept of influence supported by Henry and Mark (2003)—may have greater knowledge use and subsequent knowledge mobilization than those who have a narrow understanding of use (Donnelly & Searle, 2017; Mason & Azzam, 2019).

### Future Research Directions

Fourteen articles shared avenues for future inquiry (Adams et al., 2015; Azzam, 2011; Bourgeois & Naré, 2015; Brandon & Singh, 2009; Breidahl et al., 2017; Christie, 2007; Donnelly & Searle, 2017; Fleischer & Christie, 2009; Froncek & Rohmann, 2019; Ledermann, 2012; Mason & Azzam, 2019; Milzow et al., 2019; Vanlandingham, 2011; Whitmore et al., 2017). There is a dearth of knowledge on the levels and mechanisms of use; most of the published research examines whether particular factors influence use, but none of the articles in our review studied the shifts or steps taken following the evaluation (Bourgeois & Naré, 2015; Brandon & Singh, 2009; Christie, 2007; Milzow et al., 2019; Vanlandingham, 2011). Another area for future research is stakeholders’ perceptions of evaluation success (Whitmore et al., 2017). The need for further research into evaluation design, the impact of various data types on changing stakeholder attitudes, and the reporting preferences of different audiences were also mentioned (Christie, 2007; Mason & Azzam, 2019).

## Discussion

To keep pace with practice evolutions evaluation, use an important area for evaluators and evaluation scholars to investigate (King & Alkin, 2019). When examining evaluation use, terminology and specificity are ongoing concerns to the field of evaluation (King & Alkin, 2019). Our review reveals continued efforts to define use, influence, and utilization as the three most frequently identified terms. We share Alkin and King's (2016, 2017) perception that use and utilization continues to be ambiguous language that which encompasses and espouses ideas with persistent vagueness in practice and theory. Further research on evaluation use benefits from including and defining selected terms as well as increased specificity with the levels and mechanisms established for understanding or measuring use.

Previous reviews recognize evaluation use and utilization as dynamic and interrelated concepts (Cousins & Leithwood, 1986; Johnson et al., 2009). Studies related to evaluation use and utilization have continued to increase at a steady rate since the last scoping review (Johnson et al., 2009), with 63.8% of the 47 articles in this study published since 2014. Similar to previous studies, our sample drew upon English language publications from a select set of databases and journals. We acknowledge that additional perspectives about evaluation use and utilization (e.g., Rogers & Malla, 2019) might be gained if the language or search criteria were broader. A future direction involving texts beyond journals and a focus on worldwide perspectives would yield important new insights. Our study extends these studies by recognizing expansions in the roles and engagement of stakeholders, distilling five factors influencing use, and advocating for evaluation that responds to complex, diverse, and evolving contexts.

The vast possibilities for evaluation practice and theory means that promoting evaluation use and utilization can involve many stakeholders, take many forms, and happen in many ways. Our review shows that promoting use requires paying attention to stakeholders as well as understanding the evaluation situation. This in turn aligns with an established practice of developing relationships that encourage responsive stakeholder engagement appropriate for context (Adams et al., 2015; Breidahl et al., 2017; Christie, 2007; Donnelly & Searle, 2017; Lawrenz et al., 2011; Ramírez et al., 2017; Whitmore et al., 2017). Evaluators who spend time acquainting themselves with the culture of the program and/or organization as well as getting involved with the stakeholders may understand and be positioned to respond to ways culture influences evaluation use and utilization.

While the field of evaluation defines stakeholders as those who are invested, our review specifies those stakeholders representing only a small sample of possible stakeholders. This limited perspective is surprising, given what we know about both the potential to engage stakeholders (Cousins, 2020; Shulha et al., 2016), the value of reflection (Canadian Evaluation Society, 2018, 2020; Yarbrough et al., 2010), and the importance of recognizing diversity (Boyce & Chouinard, 2017; Hood et al., 2015). Future studies which incorporate multiple and varied stakeholders are needed to determine the reach of evaluation use and implications of evaluation utilization. Additionally, engaging broadly in research on evaluation, which may include sampling from multiple groups and widening to include community or program beneficiaries may illuminate a deeper understanding about the use or success of evaluation.

Stakeholders is a noteworthy concept, which appears in all five factors identified in this review in addition to being featured as its own factor. Another factor, resources was established as the first factor also underpinning other factors, as resource limitations influence decision-making and was explicitly stated in a few studies (Donnelly et al., 2014; Vanlandingham, 2011). The assumption in the literature analyzed for this review appears simplistic: more resources lead to greater use (Adams et al., 2015; Donnelly & Searle, 2017; Milzow et al., 2019). We suggest future research, adopting a more complex view would more fully represent the role of resources and its relationship to stakeholders and evaluation use.

The factor evaluation characteristic aligns with findings from prior reviews which demonstrates that the quality of the evaluation continues to matter; studies show that an evaluation must be high quality—and perceived as high quality—to facilitate use. Quality is determined through a multifaceted approach that considers the evaluation's design, management, and reporting (Azzam, 2011; Froncek & Rohmann, 2019; Ledermann, 2012). Evaluations with a clear purpose and novel findings are desirable. This finding is particularly salient when considering the intersections of evaluation use with knowledge work (e.g., knowledge translation and knowledge mobilization), which strives to meet the needs of various audiences (Donnelly & Searle, 2017). Quality evaluation includes facilitating for use and utilization by utilizing or developing a responsive evaluation approach that balances evaluation quality with identifying and acting on stakeholder needs.

Positioning an evaluator as a facilitator who is aware of and attentive to the social and political landscape (Ledermann, 2012; Whitmore et al., 2017). Such an evaluator is more likely to be successful in promoting use if the context is also a learning environment where relationships are responsive to changing conditions. Studies in our review show that stakeholder characteristics influence the types and usefulness of data (Christie, 2007; Mason & Azzam, 2019; Rogers et al., 2019). Evaluators can foster stakeholder engagement by teasing out assumptions about evaluation, building excitement, enhancing evaluation literacy, and explicitly interweaving capacity building to understand evidence generated in an evaluation as well as increase to the potential use of evaluation findings. Moving forward, we recognize that multiple factors are interconnected in understanding and promoting evaluation use. We encourage future research on evaluation that begins to capture the interplay of the factors in promoting use and utilization.

Emphasizing cultural competence relates to the factors identified in this review through the need for responsiveness to people and programs. This focus is likely to be amplified in future research to enhance understanding about evaluation use and utilization. Future scholarship points to the need for development of more complex theories of evaluation use. Such theories could help the field to better understand and predict the impacts of stakeholder traits, reporting styles, social relationships, and participatory processes on evaluation use (Breidahl et al., 2017; Christie, 2007; Donnelly & Searle, 2017; Fleischer & Christie, 2009; Froncek & Rohmann, 2019; Ledermann, 2012). Additionally, complex theories of evaluation use may be better able to provide insights into how evaluators are responding to power imbalances, navigating culture, and attending to equity. We suggest that case studies of instances of high and low evaluation use may be a worthy addition to the literature. These case studies would benefit from being longitudinal, multisite, and mixed methods using metrics to measure evidence of use (Poth & Searle, 2022).

Many evaluators embrace their roles as capacity builders, innovators, and change-makers who work alongside social champions to address complex challenges and use evidence for decision-making. Advancing useful evaluation in ways that promote inclusivity, equity, and fairness is an important aspect of evaluators’ theoretical and applied practice (Yarbrough, 2017). Cultural competence is a requirement for a practicing evaluator (e.g., American Evaluation Association, 2011; Canadian Evaluation Society, 2018), and cultural responsiveness is considered sound evaluation practice. Stakeholders and communities are the experts in understanding and proposing solutions; therefore, they are critical to promoting equity and fairness in evaluation (Brandon & Fukunaga, 2014). Embracing equity and fairness with stakeholders in both evaluation and research on evaluation is about working together to promote evaluation use as a contributor to the trajectory for social change (Patton, 2020a). The pathway for a more just society is strengthened when the capacity for evaluation and the research on evaluation are furthered by collaborating, embracing diverse knowledge, and working reciprocally with communities (Poth & Searle, 2017). Future research can explore the intersections of evaluation use and utilization with a focus on equity, diversity, and culture.

## Conclusion

Our use of a five-stage process (Arksey & O’Malley, 2005) with a two-stage screening, brought us review 47 studies (*n* = 22 theoretical; *n* = 25 empirical). Our review in the area of evaluation use and utilization contributes in three important ways. First, we capture important evolutions in the roles and importance of stakeholder engagement and use. We note increased recognition of the value of iterative and of intentional opportunities for stakeholders. Secondly, we identify five interrelated factors which underpin evaluation decision-making and processes. We recognize the essential role of these factors in promoting use and utilization. Finally, we advance the need for greater attention to culture and equity when considering use and utilization. We acknowledge that the lack of attention to culture and equity may be hindering our ability to understand and capture evidence in a way that honors and respects the expanded purposes and contexts for evaluation.

## Supplemental Material

sj-docx-1-aje-10.1177_10982140241234841 - Supplemental material for Mapping Evaluation Use: A Scoping Review of Extant Literature (2005–2022) Supplemental material, sj-docx-1-aje-10.1177_10982140241234841 for Mapping Evaluation Use: A Scoping Review of Extant Literature (2005–2022) by Michelle Searle, Amanda Cooper, Paisley Worthington, Jennifer Hughes, Rebecca Gokiert and Cheryl Poth in American Journal of Evaluation

sj-docx-2-aje-10.1177_10982140241234841 - Supplemental material for Mapping Evaluation Use: A Scoping Review of Extant Literature (2005–2022) Supplemental material, sj-docx-2-aje-10.1177_10982140241234841 for Mapping Evaluation Use: A Scoping Review of Extant Literature (2005–2022) by Michelle Searle, Amanda Cooper, Paisley Worthington, Jennifer Hughes, Rebecca Gokiert and Cheryl Poth in American Journal of Evaluation

## Notes

1. Reed and Rudman (2023) recognize controversy over the term stakeholder because of connotations linked to possession and property; given our retrospective analysis of the literature, we have continued to use it for this review.
