# Supplementary material for: Mapping Evaluation Use: A Scoping Review of Extant Literature (2005–2022)
Source: Am J Eval. 2024 Mar 13;45(3):341–60. doi: 10.1177/10982140241234841 (PMC11469954; doi:10.1177/10982140241234841)
Supplement: sj-docx-1-aje-10.1177_10982140241234841 - Supplemental material for Mapping Evaluation Use: A Scoping Review of Extant Literature (2005–2022) [file sj-docx-1-aje-10.1177_10982140241234841.docx]

**Appendix A**

**Data Extraction Summary for 47 Articles Included in Scoping Review**

**Table A1**
*Empirical Summaries (N=25): Characteristics and Findings*

| # | Citation | Country | Study Aim | Research Design | Population | Findings | Implications | Evaluation |
| --- | --- | --- | --- | --- | --- | --- | --- | --- |
| 1 | Adams et al. (2015) | United States | “Details the  Expectations to Change (E2C) process, an interactive, workshop-based method designed to engage primary users with their evaluation findings as a means of promoting evaluation use and building evaluation capacity” (p. 243). | Case Study:  qualitative data methods | 13 direct staff and 2  program administrators participated in the case study.  Model is meant for  the evaluators to improve stakeholder use. | **a)** One year after the E2C  workshop, 12 of the participants were asked to complete an online survey to assess the extent to which the evaluation had been used.  - 100 % response rate  - overall respondents were using evaluation results one year after the workshops.  **b)** At its core, E2C is a process  of self-evaluation and the role of the evaluator is that of facilitator, teacher, and technical consultant (p. 253).  **c)** Conceptual use took the  form of improved understanding or shifting thoughts about program aspects.  **d)** 83% of stakeholders noted  increased awareness of clients’ experiences in the program.  **e)** Six stakeholders noted their  awareness of weaknesses and areas of needed improvement when providing services to survivors.  **e)** 42% of stakeholders  increased understanding of empowerment theory and how to USE this theory when delivering service.  **f)** 54% of stakeholders at the  organizational level described using findings to add to or improve comments of the program.  **g)** 54% of stakeholders noted  how they changed their personal practices in working with clients.  **h)** Process use also saw  stakeholder growth in the areas of data collection (58%) noted training directed impacted their date collect skills and interpretation and analysis. | **a)** The correlations  between stakeholder engagement preferences and use preferences aligns with evaluation utilization literature, which indicates that stakeholder engagement [facilitates] buy-in and eventual utilization of evaluation findings" (p. 386).  **b)** Methodological  design choices influence real and perceived validity and credibility of evaluation.  **c)** Methodological  diversity and flexibility are required for evaluators.  **d)** Collaboration can be  key to methodological quality.  **e)** Question centered  evaluations are superior to methods driven. |  |
| 2. | Alkin & King  (2017) | United States | Determining a  functional definition of evaluation use and misuse, expand concept of evaluation influence, presented 4 categories that research has linked to evaluation use. | Review | N/A | No new findings or theories |  |  |
| 3. | Azzam (2011) | United States | To understand how  evaluator’s background characteristics relate to their evaluation design choices (p.376). | Survey: scenario  based with follow up questions where evaluators rated their probability of methodology preference. | Using the American  Evaluation Association (AEA) directory as the population pool, approximately 1,500 evaluators were randomly selected. Evaluators were then contacted via e-mail and asked to complete the survey only if they had k-12 evaluation experience. | **a)** An even split of evaluators  concerned with use and those who are not.  **b)** Evaluator beliefs about use  influences stakeholder engagement.  **c)** Evaluator preference for us  influences methodological decisions related to evaluation. | **a)** Stakeholder  engagement facilitates buy in and evaluation use of findings (p.386).  **b)** A direct and indirect  relationship exists between evaluator preferences and evaluation design decision.  **c)** Evaluator openness to  methodologies can promote collaboration with others who have skills and training. |  |
| 4 | Baughman et al.  (2012) | United States | Examines how the  field-based extension educators (i.e., program staff) in four extension services use the results of evaluations of programs that they have conducted. | Quantitative  Interviews with administrators, and a survey sent to informal educators | 730 informal  educators across 4 extension services respondents  4 Administrators  form the extension services | **a)** Few Programmatic changes  as a result of evaluation findings among the non-formal educators surveyed.  **b)** Extension educators tend  to use evaluation results to persuade others about the value of their programs and learn from the evaluation process.  **c)** Evaluation use is driven by  accountability measures with very little program improvement use. | **a)** Persuasive evaluation  informs others, communicates value, and demonstrates return on investment.  **b)** Extension educators  can use evaluation to learn about their programs.  **c)** Emphasis on positive  results can limit changes to programs based on evaluations.  **d)** Value exists in non-  formal educators conducting evaluations; however, support/education may be required to lead to programmatic change.  **e)** Attitude toward  evaluation can positively influence evaluation engagement.  **f)** Stakeholder  involvement in all aspects can positively affect evaluation implementation. |  |
| 5 | Bourgeois & Naré  (2015) | Canada | To investigate the  extent to which program evaluation is used in the Canadian federal government for budgetary management purposed (p.60) | Mixed methods  Organizational case  study: document review | Two government  agencies | **a)** Overall evaluation reports  clearly outline the evaluation questions and methods leading to finding and recommendations.  **b)** Key components of  instrumental utilization include:  1. Evaluations are viewed  to be credible sources of information.  2. Triangulation of data  sources and methods provide support for findings.  3. Integration of key  stakeholders provide support for findings.  4. High rate of agreement  is found in management responses which suggest the findings are relevant. | **a)** Need to extend  research beyond instrumental use  **b)** Seek to extend research  beyond evaluation processes and the usability of reports. |  |
| 6 | Brandon &  Malkeet (2009) | United States | To review a broad  sample of empirical studies of the use of program evaluation findings, not to conduct a comprehensive review (p.124) | Mined the reference  lists of five major evaluation use papers  Filtered through  355 items for empirical students that used the term “use” or a derivative.  Deep review  occurred with 52 articles | N/A | **a)** Four types of methods or  designs (surveys, case studies, simulations, narrative reflections) are used equally across the research examined with most of the studies occurring in the education field.  **Barriers and challenges:**  Studies provide more information about the relationship between evaluation features and evaluation use than they do about the level of use achieved. | **a)** Not reporting on  validity processes reflects poor methodology.  **b)** Findings need to be  linked to practical implications.  **c)** Mixing methods in one  context i.e., education and achieving sound results does not mean the methods can generalize to other contexts.  **d)** There is a need to  address content related validity as well as reliability.  **e)** The utility of evaluation  knowledge in education may trump accuracy. |  |
| 7 | Breidahl et al.  (2017) | Denmark | “To fill the (large-  scale public-sector reform research) gap in the literature” (p. 226)  “We argue that not  only is knowing how to evaluate large-scale public-sector reforms of critical importance; so too is knowing how to conduct research on and evaluate the nature of these evaluations, their utilization, and the broader role they play in public-sector reforms” (p.227). | Mixed methods:  literature review, comparative analysis of two evaluations across two phases. | N/A | **a)** Despite the complex nature  of large-scale public sector reforms, the authors demonstrate an understanding of evaluation in this context.  **b)** Despite the two reforms  being similar there are differences in how programs organized and emphasized the evaluations i.e., internal versus external approaches.  **c)** Evaluation processes and  utilization are influenced by the methods used to organize and carry out the evaluation.  **d)** The role of the evaluator is  influences by the internal and external nature of the process selected.  **e)** Contextual factors such as  understanding the institutional cultural perspectives can influence evaluation processes and results.  **f)** “The 'chaos perspective’  contributes to understanding of evaluation processes as emergent rather than linear and predictable” (p. 240). | **a)** External evaluators can  play role as change leaders by facilitating stakeholder dialogue, listening to demands for usable knowledge.  **b)** External evaluators  need to continuously focus on building trust (because usability is not anticipated). Internal evaluator results are not considered trustworthy or independent but can be powerful for stakeholders.  **c)** Academic evaluators in  this context must actively engage in trust building activities.  **d)** Creating facilitation  processes to discuss and handle reform ambiguity would benefit academic evaluators. |  |
| 8 | Christie (2007) | United States | Examine the first  level of influence, the individual…to contribute to literature on evaluation influence which can be examined collectively to develop empirical literature on the evaluation process. | Online surveys,  simulation studies | Participants in  leadership roles from two academic educational leadership degree programs | **a)** Participants are likely to use  large scale study data, case study data, and anecdotes equally.  **b)** Educational background,  sector of employment, and the degree to which decision makers were influenced can impact the degree to which data influenced participants. | **a)** “It may be valuable to  also consider the influence that evaluation information may have on individuals’ beliefs” (p.22).  **b)** “Findings may offer  insight into the types of information that influence the individuals involved in evaluations more generally… findings of this study would also apply to other important program stakeholders, for example program staff” (p. 22).  **c)** Different information  influences participants’ behaviour in different ways which may be likely that influencing behaviour can influence attitudes.  **d)** There could be an  advantage in evaluators directly to identify evaluation information that could be most influential based on social and political contexts.  **e)** “Evaluators should not  dismiss the influence of anecdotal evidence” (p. 21). |  |
| 9 | Clinton (2014) | Australia | "This study sought  to find confirmation that stakeholder engagement in evaluation could influence the outcomes of a program and thus subsequently provide evidence as to the merit and significance of evaluation engagement" (p. 121). | Case study of two  programs using a variety of methods including reports, surveys, interviews, observations, and secondary data. | Program  stakeholders | **a)** Stakeholders’ engagement  in evaluations provided reasonably unique contributions to the overall program outcomes.  **b)** Evaluation readiness, and  collaboration are all good predictors of sustainability.  **c)** Too simplistic to suggest  that an outcome can be achieved through an intervention activity alone—as a complex mixture of constructs come into play. | **a)** "The model is able to  provide a clear picture of the place of evaluation in bringing about sustainable change...[and] provides the impetus for ECB" (p. 126).  **b)** Evidence that  evaluation engagement and use of evaluation are linked.  **c)** Evaluation can be a  contributor to social change.  **d)** Evaluation appears to  play an important role in ensuring that organizational processes and collaboration are in place to promote program sustainability after the evaluation. |  |
| 10 | Donnelly et al.  (2014) | Canada | To examine  knowledge translation int eh context of evaluation with the evaluator as the knowledge broker | Single case study  design during an evaluation | Evaluator as  knowledge broker  Memory clinic  stakeholders | **a)** Context has a strong  influence on knowledge translation needs.  **b)** Supporting knowledge  translation activities throughout the process – can include weekly e-newsletter, monthly process meetings, maintaining a presence.  **c)** Capacity building of  stakeholder to engage in knowledge translation includes identifying and applying knowledge within the service delivery of the program.  **d)** Knowledge translation can  support the evaluation if it occurs throughout the evaluation.  **e)** “Conceptualizing evaluation  as a change process as well as an approach to measure change opens the door for evaluation to be considered as a mechanism of knowledge translation” (p.53). | **a)** Evaluators need to  have content knowledge about the program being evaluated in order to enmesh external knowledge.  **b)** Evaluators will take on  a brokering role when doing knowledge translation informed evaluations, this means evaluators will need skills to be able to respond to the knowledge needs of the organization.  **c)** Knowledge translation  focused evaluators will need to continue developing their understanding of the program evaluation standards (Yarbrough, 2011).  **d)** The view of evaluators  adding the knowledge translation role will either be viewed as beyond their capacity or an ideal method.  **e)** Evaluators will need to  consider taking on the knowledge translation role as the research is shifting toward including this in the evaluation work – however capacity must be built. |  |
| 11 | Fleischer &  Christie (2009) | United States | Three foci:  1.To examine  evaluators perceptions and experiences related to use theory  2. To discover how  survey data from the current study compares to Preskill and Caracelli (1997)  3. To identify  characteristics that distinguish high endorsers of use items from others in the sample. | A mixed-methods  cross sectional survey design | Members of the  American Evaluation Society | **a)** Respondents of both  surveys noted stakeholders should be involved in the evaluation process to influence use  **b)** Role of the evaluator can  influence use  **c)** Evaluation activities can  influence organization learning  **d)** Yet 68% of the overall  sample report non-use of evaluation as a problem | **a)** Descriptive studies help  draw an outline of the field and those who practice evaluation.  **b)** Practitioners gain  insight into the attitudes and opinions of other practitioners, which act as a point of comparison for one’s own beliefs, thereby encouraging personal reflection, which is key to forming notions of identity. | Unique evaluation  framework developed – see Key Lessons |
| 12 | Froncek &  Rohmann (2019) | Germany | “The aims of the  current article are to assess the possible negative effects of participatory evaluation on evaluation use and to better understand the psychological mechanisms that might potentially underlie participatory evaluations effects” (p. 20). | Quantitative: “Two  online studies with different samples are reported, both using experimental vignette” (p.22).  “Participants were randomly assigned to one of three conditions (participatory, pseudo-participatory, and non-participatory evaluations) and asked to complete a survey after” (p. 23). | One sample is  university students  The second sample  is practicing social workers | **Barriers and challenges:**  **a)** “pseudo-participatory  evaluation clearly reduced reported instrumental and conceptual use compared to participatory and non-participatory evaluation” (p. 25).  **b)** Due to the frustration  effect justice perceptions had a negative indirect effect on evaluation use. | **a)** “It might be wise for  evaluators to become sensitive to stakeholders’ perceptions of fairness in participatory evaluation processes, just as evaluators need to be concerned about relationship quality, trust, or power and authority” (p. 28).  **b**) “Evaluators are advised  to facilitate positive interdependence and cooperative goals in order to develop promotive interaction” (p. 29).  **c)** Scholars are well  advised to consider stakeholders’ socioemotional interests in addition to their instrumental interests when conceptualizing and conducting participatory evaluation and when trying to understand and account for the associated processes” (p. 29).  **d)** “The authors point to  Shulha et al. (2015) CAE principles to encourage effective participatory evaluations and ‘avoid pitfalls and backlashes’” (p. 29). |  |
| 13 | Johnson et al.  (2009) | United States | Empirical review of  the literature between 1986-2005 | Systematic review of  the literature | 321 Abstracts  screened  98 Publications  screened  47 Articles  comprised initial analysis | **a)** “Two categories and 12  characteristics from Cousins & Leithwood (1986) still hold with an even spilt across - evaluation implementation and the others about decision making and policy setting. Characteristics from each of these categories were all examined in at least one article - the most prevalent characteristics was communication quality” (p. 381).  **b)** A main change in use was  identified by Shulha & Cousins (1997) related to evaluator competence.  **c)** Stakeholder involvement is  another category added to the original review. There are increased participatory approaches with multiple names; they identified 9 characteristics. 23 of 41 studies addressed stakeholder involvement related to use. | **a)** Process use is still a  rather recent concept that does not have a large body of strong literature. The field of evaluation is still more focused on outcomes and results. |  |
| 14 | Larsson (2021) | Sweden | “To investigate to  what extent and how the national evaluations and evaluation system are used by key actors and how they contribute to (sustainable development” (p. 454). | General qualitative  design: document review, observations, interviews | NEQO policy  documents and meetings, 21 interviews with key actors (central, proximal, peripheral to national policy cycle). | **a)** All central actors claimed to  use evaluations compared to a single peripheral actor.  **b)** Instrumental and  conceptual use are most common.  **c)** Central and Proximal actors  are more likely to use evaluations than peripheral actors.  **Barriers and challenges:**  **a)** Different factors lead to  non-use dependent on actor’s role.  **b)** Central actors needed the  evaluations to be relevant, credible, timely and part of their work tasks to use them.  **c)** Proximal actors needed the  evaluations to have the same qualities but also stakeholder engagement.  **d)** Central actors’ non-use  increased with limited analysis that doesn’t take enough context into account – peripheral actors cite the same in addition to missing capacity building and inaccessibility of evaluation. | **a)** Stakeholders should be  involved to increase use, particularly for peripheral actors.  **b)** Evaluations focusing  on environmental factors need to analyze with a more careful and expansive understanding of societal context. |  |
| 15 | Lawrenz et al.  (2007) | United States | “To provide a  reflective case narrative of a program evaluation in which a wide variety of dissemination techniques were employed because of the changing context within which the evaluation operated” (p. 277). | Mixed met Surveys -  web based Site Visits - 13 different ATE projects, originally intended to be used as a case study. Ended up being used in a set of issue papers related to ATE program features. | Staff in the ATE  Programs | **a)** Different dissemination  techniques can be used to reach different audiences and to address different needs of the same audience. Techniques include fact sheets, fully reports, trifold brochures, site handbook, issue papers, and a hyperlinked electronic synthesis of the video conference.  Each one was shown to benefit a stakeholder group more than another technique thus all are useful across contexts. | **a)** “Evaluation Trade off”  (p. 287) - evaluation teams expedite information sharing to stakeholders - this may lead to some inaccuracies. Therefore, balance timeliness of reporting with ensuring review of data by qualified stakeholders is a factor to be considered in evolution. |  |
| 16 | Ledermann (2012) | Switzerland | “This article  examines the interplay between the policy and decision setting on the one hand and specific characteristics of evaluation implementation on the other hand” (p. 163). | A qualitative,  comparative case study design. | 14 desk managers  were interviewed | **a)** “No single condition alone  is necessary for the occurrence or non-occurrence of an evaluation-based change decision” (p. 169).  **b)** “In evaluation as awakener  scenarios the two conditions of novelty and high quality are necessary for an evaluation to trigger change in a consensual situation with a low pressure for change” (p. 86).  **c)** “In evaluation as trigger  scenarios an evaluation must be conceived as good quality in order to trigger change in consensual context with high pressure for change and that novelty is less of an issue” (p. 171).  **d)** “In evaluation as referee  scenarios decisions were made according to the truth test…high quality evaluation is only necessary if an evaluation challenges pre-existing beliefs of decision makers” (p. 171).  **e)** Perceptions of quality and  methodological rigour are identified as more important than previous thought.  **f)** An evaluation’s quality and  novelty influence its use in decision making. | **a)** “Teachers’ social  networks are more amenable to outside influence than previously thought” (p. 331).  **b)** “Social policy can play  a role in fostering conditions in schools within which teachers seek out their colleagues, share information, solve problems, and learn from one another in their networks” (p. 331).  **c)** “Understanding the  role of teachers’ social relations in individual change, organizational processes, and student outcomes may require greater attention to the ways that social networks are, themselves, embedded in and affected by their organizational and policy context” (p. 331). |  |
| 17 | Marra (2021) | Italy | To “provide  behavioral insights on Italy’s performance regimes and suggest a behavioral design to reform Italy’s evaluation policy” (p. 485) | Qualitative:  4-year Ethnography - observation of two sites and interviews | Various actors in  Italy's governance systems | **Barriers and challenges:**  **a)** Evolution of Italy's  evaluation policy is mostly in response to mandatory requirements from the European Union - top-down  **b)** Because it is done in  compliance, it is not really integrated or embedded with performance measures or other uses.  **c)** Evaluations, particular of  employees, are not used for interpersonal reasons.  **d)** Hesitation to use  evaluations in politically charged contexts - people want to keep their jobs.  **e)** Evaluations are skewed due  to gender biases present in workplace.  **f)** Evaluation findings are  rejected when they challenged the status quo.  **g)** Systems built on rewarding  those already in power do not encourage evaluators to challenge the system.  **h)** It is up to individual  professionals to be brave enough to start these conversations.  **i)** Fear of evaluation can lead  to misrepresentation of data. | **a)**"Cultural separateness  [of adjacent disciplines] contributes to undermining the credibility of evaluation within the organization, reinforcing the bureaucratic compliance mentality against evidence use" (p. 496).  **b)** "Partisan leadership  undermines the capacity of governments to use evidence effectively" (p. 497).  **c)** Ineffective managerial  accountability can ruin productive work and evaluation cultures.  **d)**Evaluators need to be  aware of their own implicit biases within organizations - peer review can help.  **e)** Evaluators should  adhere to professional standards so they don't give in to self-interest.  **f)** Evaluators should pick  up ethically rooted approaches to navigate challenging evaluation contexts rife with biases and compliance mindsets. |  |
| 18 | Marshall et al.  (2022) | United States | “To describe the  Project PrIDE (PrEP implementation, Data to Care, and Evaluation) through the lens of Evaluation Utilization and provide examples of how twelve funded health departments (HD) utilized evaluation findings to make decisions related to improving PrEP awareness and uptake, and/or enhancing capacity for data to care (D2C) activities” (p. 91). | Qualitative: Analysis  of final evaluation reports | Local and state  health departments | **Stakeholder perceptions:**  **a)** All health departments used  the evaluation in some way.  **b)** Most common uses were  improving strategies and advocacy/decision making.  **c)** Community engagement  followed many of the evaluations too. | **a)** Should request a  utilization plan in the evaluation.  **b)** Stakeholder  engagement is critical. | Process Evaluation;  indicators listed in article |
| 19 | Mason & Azzam  (2019) | United States | The purpose of the  article is twofold:  "(1) determining whether evaluation information can influence the general publics’ attitudes about social programs, thereby providing an empirical assessment of the individual-level attitudinal component of the Mark and Henry framework, and  (2) identifying whether one particular communication modality, data visualization, is more likely to influence the publics’ attitudes about social programs" (p. 250). | Qualitative:  implemented an online simulation of evaluation reporting. | 1,425 adult  participants from the general American public with Amazon's Mechanical Turk. | **a)** Stakeholders' global  attitudes towards the program tend to follow the "direction of the data" (p. 257).  **b)** "readers respond to  evaluation findings in different ways based on their initial attitudes toward the evaluand" (p. 259).  **c)** Findings suggest that  readers examining visual reports with ample data visualization hold slightly less positive attitudes toward evaluation and the report than individuals who read reports without data visuals (p. 260). Further, individuals who received reports with visuals perceived the reports to be less credible and harder to understand. | **a)** "Initial attitudes—  although not attitude certainty—were shown to predict attitude change above and beyond report content. However, this latter, unexpected finding may be explained by the MTurk sample’s lack of vested interest in the evaluation findings" (p. 262).  **b)** The study provides  evidence that reinforces "one hypothesized pathway in the Mark and Henry's (2004) model of evaluation influence" (p. 264).  **c)** Evaluators wishing to  embed data visuals into their reports ought to prepare audiences to understand and interpret the visuals. Data visuals in reports can have negative impacts on readers' perceptions of the report and evaluation; this may be influenced by lack of familiarity with data visuals, the requirement to analyze information to arrive at conclusions, and the perception that visually designed reports contain less detail than non-visual reports (p. 262-263).  **d)** "It is possible for evaluators to shape reader attitudes through evaluation reports" (p. 263).  **e)** "Readers respond to  differently to evaluation findings based on initial attitudes and the report’s degree of consistency with those attitudes" (p. 263).  **f)** "Evaluators should be  cautious about overestimating the effects of data visualization on readers’ responses to evaluation reports" (p. 263).  **g)** "It becomes essential  for evaluators to consider readers’ prior attitudes when preparing reports. In this way, it also suggests evaluators may need to broaden their repertoire of communication strategies beyond mere reporting of results to also include techniques that might be more persuasive when counteracting ideologically entrenched views" (p. 263). |  |
| 20 | Milzow et al.  (2019) | Switzerland | “To understand the  role of evaluation and use this knowledge to make evaluation worthwhile within the field of research evaluation and hopefully also in a broader context” (p. 95). | Qualitative:  Research on evaluation that accessed 11 publicly available evaluation studies.  Case study,  interviews, written material | 25 individuals who  had participated in one of 11 evaluation studies previously completed represented in the Science Europe Working Group. | **a)** Varying uses of evaluations  (co-) exist, a hierarchy of use is found in the case studies.  **b)** Steering use (supported  decision making or policy change) and information use is most widespread – Learning use followed and the Legitimizing (justify funds) and Ritual use (systematic evaluations done mainly for research institutions.  **c**) “Context factors and others  external to the evaluation influence the use of evaluation” (p. 100). | **a)** “Since evaluation uses  are multiple and depend on a number of interrelated factors, we would like to suggest the application of logical models to the use of evaluation itself” (p. 100).  **Questions of interest include:**  **b)** “How does having a  pre-defined management response process or a reflection team accompanying the evaluation influence the way studies are used?” (p. 100)  **c)** “In which way does the  hierarchical level at which an evaluation is ordered, or the position of the staff included in the discussion process determine the commitment to the recommendations?” (p. 100) |  |
| 21 | Ramírez et al.  (2017) | Canada | Analyze a case  study based on empirical experience with the lens of evaluation use and influence. | Analysis of case  study | Members (staff) of a  youth training employment program | **a)** evaluation allowed the  funder to make its decision to renew the partnership with CAP YEI.  **b)** Findings demonstrate how  and why the BEST model was achieving outcomes and highlighted challenged - BEST model’s non-negotiable components were kept during the expansion of the program.  **c)**Findings demonstrated how  to achieve the desired results-thus helped to design the next phase.  **d)** Results have been noticed  by partners who will use the UFE findings in presentations to influence policy and to increase the profile of CAP YEI.  **e)** The UFE experience has  been shared widely as both the process and the results benefitting the program this has led to:  - brand recognition - credibility among peers, funders, government agencies - papers and presentations have been shared | **To answer the research questions**   **a)** Enabling factors for  funders/grantees to turn evaluation into a learning intervention include:  - explicit review of partner roles - a range of evaluation purposes aside from accountability  - early exposure of the evaluation 'owners' to the findings p.19 - opportunity for evaluation owners to influence emergent conclusions  **b)** Features of UFE clarify  projects theory of change include:  - made clear their role was not authoritarian, rather as story tellers and researchers - moved beyond neutral evaluator role and asked tough questions to uncover assumptions  -noticed enablers such as ratio of trainers to students that allowed instructors to increase positive relationships -began to flag possible limits of the model in different contexts i.e. urban vs. rural -began to expand the theory of change by adding in more detail  **c)** How UFE bridges  between summative view of achievements and developmental contribution to future work:  - UFE demonstrated the achievements of the program were confirmed and triangulated and therefore the reason BEST model received validation  - process allowed for iterative feedback of emerging findings which build a partnership between evaluators and clients/project managers - what began as a summative evaluation led to learning opportunities which turned into a phase two - Developmental Evaluation. |  |
| 22 | Rogers &  Gullickson (2018) | Australia | “Explores  champions in organizational settings and highlights the need for increased understanding of evaluation champions” (p. 46). | A systematic search  of selected databases | Relevant articles to  the topic | Authors note the main  findings related to activities of evaluation champions.  **Evaluation champions may:**  **a)** Advocate for support and  resources.  **b)** Motivate others, provide  energy, interest, and enthusiasm.  **c)** Provide access tools,  resources, networks, and expertise.  **d)** Help others to apply  evaluative thinking, use evaluation findings and create opportunities for reflection.  **e)** Assist, train, mentor,  support evaluation while considering different perspectives and encouraging others to contribute.  **f)** Consider how evaluation  can be strategically promoted and sued for organizational change.  **g)** Ask and encourage others  to ask critical questions and initiate discussions and debates.  **h)** Develop engaging ways to  explain details and develop common visions. | **Suggestions for**  **improvement:**  **a)** Promote can recognize  champions, and work to support a more operationalized view of champions skill set.  **b)** Mobilize the  knowledge related to evaluation champion roles and potential influence on program evaluations.  **c)** Increase recognition of  the extent that evaluation champions can benefit evaluation. |  |
| 23 | Rogers et al. (2019) | Australia | “To stimulate a  wider conversation and further advance understanding of evaluation literacy by exploring the role of evaluation literacy in internal evaluation in the non-government sector" (p. 2). | Qualitative: The  authors used narrative inquiry combined with autoethnography to explore their experiences in the under-researched area of internal evaluation and evaluation literacy in the NGO sector. | The authors  themselves - three Australian evaluators undertaking doctoral studies with an emphasis on NGOs and internal evaluation. | **a)** “These auto-narratives  highlight the importance of evaluation literacy, as they demonstrate the multiplicity of roles that internal evaluators play in building evaluation literacy to maximize the use of evaluation" (p. 13).  **b)** "The auto-narratives reveal  some of the underlying issues affecting evaluation use in NGOs, such as findings not being communicated or used for decision making, opportunities for learning not being incorporated, evaluation reports not being appropriate for the target audience, and suspicion and anxiety being shown toward evaluation and its intended purpose" (p. 13).  **c)** "Internal evaluation practice  therefore, requires high-level cognitive and social skills to motivate and engage colleagues to access, understand, and use evaluation information. Humility, rapport building, passion, humour, persuasion, and overt and covert facilitation and persuasive and influential communication were some of the interpersonal qualities noted in the narratives" (p. 13).  **d)** Internal evaluators are  positioned well to increase evaluation literacy and use by using participatory methods to encourage evaluative thinking and use within the organization.  **e)** Building the capacity of  participating researchers, practitioners, practice organizations, and research organizations to engage in partnership work. | **a)** Internal evaluators may  view external evaluators "as critical friends" (p. 15).  **b)** Internal evaluators may  seek tertiary training in evaluation (p. 15).  **c)** Internal evaluators may  build strong relations with organizational staff (p. 15).  **d)** Internal evaluators may  maintain a reflective mindset (p. 15).  **e)** Internal evaluators may  "[seek] to diminish evaluation anxiety through engaging with staff as peers and using humour" (p. 15).  **f)** Quantitative measures,  while time effective and minimally disruptive to practitioners’ work, are challenging to develop (e.g., due to the lack of common language between partners).  **g)** The purpose and  audience of the assessment need to be a primary consideration. |  |
| 24 | Vanlandingham  (2011) | United States | To examine the  methods used by legislative evaluators to interact with their key stakeholders (elected legislators and their staff) and report evaluation | Quantitative:  Two nationwide surveys were administered | 42 State Legislative  Offices | **a)** “Substantial variance in  legislative use of actions recommended in the literature “particularly those that required extensive resource commitments and interactions with legislative stakeholders” (p. 89).  **b)** “Evaluators that more  proactively took steps were viewed as having more value and impact in the legislative process than those who made less effort” (p. 89).  **c)** “The precepts of utilization  scholars that stress the importance of forming strong network linkages between evaluators and the intended users of their work as well as the importance of providing readily actionable research products” (p. 92).  **d)** “Differing normative  guidelines reflect the tension between evaluator’s desire to provide objective findings and their desire to provide information that is useful to their clients” (p. 96). | N/A |  |
| 25 | Whitmore et al.  (2017) | Canada | "In the present  study, we were interested in how evaluators frame success in CAE" (p. 332).  CAE – Collaborative Approaches to Evaluation. | Completed online  instrument that included quantitative and qualitative items. | 320 practicing  evaluators | a) “Evaluators define the  success of CAE in terms of specific evaluation consequences, that is, direct use of evaluation findings" (p. 335).  b) “CAE influences process  use through a number of factors, including intentional evaluation capacity building, use of findings, and transformative effects” (p. 336).  c) “embedding stakeholder  engagement throughout the CAE projects has the possibility of increasing process use and, ultimately, the use of findings" (p. 340).  Barriers and challenges:  “The non-use of evaluation  data could be taken to define observed lack of success. We observed a political and/or non-rational element to this discourse" (p. 335). | a) Propose a conceptual  framework that represents the prevalence of and the low, moderate, or high evaluation aspects and their impact on evaluations use both use of findings and process use. |  |

**Table A2**
*Theoretical Article Summaries (N=22): Characteristics and Findings*

|  | Citation | Country | Study Aim | Research Design | Population | Findings | Implications | Evaluation |
| --- | --- | --- | --- | --- | --- | --- | --- | --- |
| 1 | Alkin & King  (2016) | United States | Review the historical  evolution of the concept of use. | N/A | N/A | Evaluation use has historically been explored through:  **a)** An educational measurement perspective where the purpose is to provide decision makers with information.  **b)** A social science research lens where knowledge can be used to inform decision making. The three commonly accepted categories of use based on evaluation findings emerged and included instrumental, conceptual, and symbolic use.  **c)** The addition of process use  has been added to the field, which acknowledges the learning that occurs through participation in evaluation activities. | **a)** Broadening  consideration about the use of evaluation to the organizational level expanded the concept, including thoughts on how to create evaluation capacity through the evaluation process. |  |
| 2 | Amo & Cousins  (2017) | Canada | The authors revisit  arguments supporting inquiry that takes up the challenge of connecting the cognate fields of evaluation utilization and the broader domain of knowledge utilization (p. 81). | N/A | N/A | **a)** By means of a thorough and  well-articulated conceptual essay and the description of several case examples, the authors have highlighted the parallels between evaluation utilization and knowledge utilization research and have raised issues of importance to evaluation theory and practice. | “By treating evaluation  results as a particular form of knowledge, one that competes against other forms of knowledge – both explicit and tacit- for the attention of decision makers and knowledge users, we continue to ensure the development of more effective ways of achieving the goals of our practice” (p. 84). |  |
| 3 | Appleton-Dyer et  al. (2012) | New Zealand | An analysis of key  concepts is used to develop propositions about the relationships between evaluation attributes, partnership functioning and characteristics, partnership, which builds on the work of Mark and Henry (2004).   The model is by no means complete or conclusive. It highlights gaps within the field. In this respect, the model will be able to guide future research, while also contributing to current evaluation theories and practice. | N/A | N/A | The model presented  identifies:  **a)** Participatory approach to  evaluation, credibility of the evaluator, timeliness, responsiveness, and technical quality of outputs are linked to enhanced influence.  **b)** Partnership functioning  (leadership, decision making, adaptation process, and culture) between evaluators and stakeholders play a role in evaluation influence.  **c)** Characteristics such as size  and complexity of the partnership are important for evaluation influence.  **d)** Contextual elements such  as time, resources, organizational and political standing of the organization within the evaluation are also important to influence. | **a)** Evaluators using a  participatory evaluation approach can enhance evaluation influence 1b. Skillset of the evaluator to utilize "feedback that is of sound technical quality, timely, sophisticated, credible and responsive to stakeholder needs will also be more influential" (p. 542).  **b)** To influence and  engage leadership and decisions makers in the evaluation and the process of the evaluation.  **c)** Develop processes for  change to be responsive during the evaluation.    **d)** Create processes to  manage challenges or barriers that arise during the evaluation.  “Currently, however, the  evidence behind these connections is limited. The model would benefit from a greater understanding of the connections between the factors within the model. For example, it would be useful to explore whether any evaluation approaches and activities, such as participatory approaches, and evaluation capacity building could enhance partnership functioning and evaluation behavior with a view to facilitating influence” (p. 543). |  |
| 4 | Blake & Ottoson  (2012) | United States | **a)** Examines the history  of knowledge utilization.  **b)** Explores processes  of knowledge utilization i.e., meaning of use, key theories/models, contextual variables.  **c)** Compares knowledge  utilization to other processes of sharing knowledge.  **d)** Discusses knowledge  utilization lens for evaluation | A case study is  presented to solidify the points being made. | N/A | **a)** Use needs to be included in  evaluation at the onset not as a final task in the process.    **b)** Engage stakeholders early  in the process to help identify form of use for the evaluation.    **c)** Part of stakeholder  engagement is understanding the context of the evaluation and how this will influence use.  **d)** Knowledge utilization can  be reciprocal in informing other theories and models.  **e)** Knowledge utilization does  not have to be present in all processes for understanding to be valid. | **a)** “Knowledge utilization  serves as the natural starting point for evaluation” (p. 28). |  |
| 5 | Brandon (2011) | United States | This chapter is a  reflection on the findings of four National Science Foundation evaluation case studies. | N/A | N/A | **a)** The published literature on  evaluation use by and large consists of single-site studies in which on-site program personnel are the primary audiences of the evaluations.  **b)** The case studies reported  here address secondary audiences’ use of evaluation findings and the unintended effects of involvement on stakeholders’ capacities to understand and conduct evaluations (that is, process use).  **c)** Secondary users in multisite  evaluations are less likely to use evaluation findings or processes than primary users in single-site evaluations. | **a)** “Future research is  needed and possible, but the complexities noted in the case studies will continue to complicate our understanding of the relationship—a methodological and epistemological scenario that is quite familiar to seasoned program evaluators!” (p. 95). |  |
| 6 | Conner et al.  (2012) | United States | The goal of this  framework is to bring the topic of context into the spotlight of evaluation concerns. (p. 89). | N/A | N/A | **Benefits:**  Context assessment can  benefit evaluators through prioritizing assessing and reassessing context throughout the evaluation this allows for a focused approach to the particularities of the evaluation which benefits the evaluand and the evaluator.  **Barriers and challenges:**  Context assessment is not  perfect, they cannot be rigidly applied and are subject to the points of view of both the evaluand and the evaluator which can distort the process and outcomes and potentially have key factors missed. This process also requires more time in the evaluation process. | **a)** Context assessment  acknowledges the individuality of each program evaluation and offers a place to begin making context more explicit throughout the entirety of the evaluation. The framework provided by the authors attempts to provide a systematic tool to approach context. |  |
| 7 | Cook (2014) | United States | The authors outline  strategies to increase the chances of effecting community change and social justice in evaluation through a community psychology lens. | N/A | N/A | **a)** Cook states that community  psychology has identified program evaluation as a core practice competency in training and as such the AEA guiding principles are looked to by community psychologists. Through this context the community psychology field has noted that the AEA can add social justice as a primary activity of evaluation and evaluation practitioners.  **b)** Cooke notes that evaluators  who wish to effect social justice can adopt ten strategies to build into their practice:  **1.** Choose to evaluate  programs that attempt to reduce disparities, meet the needs of disadvantaged or underserved people, or empower marginalized members of society.  **2.** Help programs with  social justice goals become more effective.  **3.** Consider the community  and the disadvantaged clients of the organization as the primary client of the evaluation.  **4.** Use evaluation methods  that increase the voice of the clientele/community.  **5.** Provide and present  actionable results.  **6.** Share results in a clear,  understandable manner with those who have the power to effect change.  **7.** Share results with those  who can advocate for change.  **8.** Help disadvantages  groups become their own advocates.  **9.** Link stakeholder groups  that can coalesce to become more effective together in pushing for change.  **10.** Use the press to  publicize findings. | **a)** “The change efforts are  more likely to be successful when the organization or program possesses an ability to engage in self-criticism and processes some desire and willingness to learn and change (p.116). |  |
| 8 | Donnelly & Searle  (2017) | Canada | The aim of this article is  threefold:  **1.** To examine the  developments in evaluation use since Shulha & Cousins 1997 paper.  **2.** To explore the  knowledge fields, focussing on knowledge translation and mobilization to further refine and develop our understanding of use.  **3.** To imagine what  future research that interweaves the knowledge field with the field of program evaluation might look like and how it has the potential to serve the contexts where this research would be conducted. (p.305). | N/A | N/A | **a)** Individual and  Organizational Learning  - Evaluation capacity building can be influenced through knowledge and is a critical aspect for the growth of the field  -The field of knowledge supports the evidence that achieving use requires a conscious effort engage in strategies that are known to support use including understanding the stakeholders and the context they are represent, and that engaging in participatory focused evaluations are key to achieving use.  **b)** Evaluation Influence  - Incorporating the knowledge and use fields allows for evaluators to focus on seeking evidence that supports the operationalization of use through articulated outcomes. | **a)** Focusing on knowledge  can serve to foster use. Linking the fields can promote the concept of use in evaluation beyond what the evaluation literature says as well reciprocally with the knowledge field, it can also serve promote evaluation as systematic approach to inquiry. |  |
| 9 | Granger &  Maynard (2015) | United States | "We describe three ways  to make impact evaluations more useful to policy and practice: emphasize learning from all studies over sorting out winners and losers; collect better information on the conditions that shape an intervention’s success or failure; and learn about the features of programs and policies that influence their effectiveness" (p. 558). | N/A | N/A | No new findings | "**1.** Emphasize learning  from all studies over sorting out winners and losers. **2.** Collect better information on the conditions that shape an initiative’s success or failure. **3.** Learn more about the features of programs and policies that influence effectiveness" (p. 559). |  |
| 10 | King & Akkin  (2014) | Canada | Details the centrality of  use in current practice and examines theories of and research on evaluation use and influence (p. 431). | N/A | N/A | **a)** “To summarize, we believe  that future research should pay close attention to the evolving contexts of evaluation use and of the need for a common definition and outcomes” (p. 451).    **b)** “Use is a multifaced process  dependent on many factors some within the evaluators control, others related to settings where evaluations took” place (p. 452). | **a)** “At this time, the  question of what explicit theory might help evaluators improve practice and increase the appropriate use of the evaluations process and its results remains just that – a question” (p. 449). |  |
| 11 | Lawrenz et al.  (2011) | United States | The purpose of this  cross-case analysis is to highlight similarities and differences across these cases to shed light on the role of involvement and use in large, multisite evaluations (p. 50). | Cross case analysis  of 4 National Science Foundation case studies | N/A | Several similarities across the 4  case studies emerged - Summarized from (pp. 51-  53):  **a)** The type of interface  with the NSF influenced stakeholder involvement and use.  **b)** Life cycles of programs,  projects and individuals influenced stakeholders’ involvement and use.  **c)** Local project control on  involvement and use of the evaluation is a factor - level of control each project had over their own involvement.  **d)** Tensions between local  project goals and program evaluation goals affected involvement and possibly use.  **e)** Results more likely to be  used stakeholders perceived the work as high quality, or the evaluator as competent.  **f)** Developing a community  fostered both the involvement and use of the evaluation.  The findings introduce the  study of evaluation use and involvement by unintended users. | **a)** Methods developed to  determine the influence of the involvement in and use of evolution on unintended users.  **b)** Deliberate and frequent  communication between evaluators and stakeholders is a key aspect of influencing involvement and use.  **c)** Consider the life cycles  of individuals and the projects in the evaluation - based on this evaluators need to plan for different types of involvement and use.  **d)** In doing multisite  evaluations tensions between the overall evaluation and local projects will be found in the evaluation efforts of stakeholders.  **e)** Perceptions of  involvement are not consistent across all individual stakeholders in the projects. |  |
| 12 | Liket et al. (2014) | Netherlands | “We provide practical  conceptualizations of the central objectives of evaluations and propose a framework that can guide negotiation processes. It presents the relationships between the evaluation purpose, evaluation question, and the different levels of effects that should be measured” (p.171). | N/A | N/A | **The Framework:**  **a)** Creates a way for evaluators  to orient stakeholders to evaluations and highlights that evaluation can fulfill more than one purpose – also that the purpose can be negotiated.  **b)** Establishes clarity in  communication needed in designing/establishing the evaluation. | **a)** “Evaluations should  start with a definition of the purpose as the basis for determining the evaluation questions and level that the effects should be measured” (p. 185).  **b)** Non-profit  organizations embrace theory and implementation into their evaluations and not put as much weight on methodological rigor. |  |
| 13 | Mark (2017) | United States | “To explore the  question "How should we increase evaluation capacity regarding social disparities?” (p. 127). | N/A | N/A | No new findings | **a)** "One implicit lesson is  that when ECB efforts are undertaken in many locations, practitioner and scholarly communities will have considerable expertise and relevant local knowledge but may have limited (if any) experience with formal program evaluation." (p. 130).  **b)** “An evaluator seeking  to increase evaluation influence might think about how to increase the motivation and ability of relevant parties to engage in sensemaking about the key messages from their evaluation (such as whether the program reduced health inequalities). With further experience, training on such matters might be incorporated into future ECB efforts” (p. 134).  **c)** “Evaluators can think  about the chain of events that need to occur, and within limits of feasibility and evaluator role, evaluators can think about what they—and possibly others—can do to help the various processes take place. Again, with further experience, future ECB might attend to such matters” (p. 135).  **d)** “Planned pathways  may well need to be supplemented with more emergent influence efforts, either when the initial plan goes awry or when an unexpected opportunity arises” (p. 136).  **e)** “Moreover, for topics  as multifaceted as health inequities, any single evaluation will often be inadequate to tilt important actions. Instead, evaluators may need to seek to increase the (appropriate) influence of their evaluation, while integrating findings from other evaluations and from other evidence. They may need to think of the proper influence of evaluation, not as the responsibility of the evaluator, but as a shared responsibility of a community of evaluators and others” (p. 136).  **f)** Evaluators ought to  support deliberate spaces and consider laying out potential action’s folks can consider when they learn about evaluation findings (p. 136). |  |
| 14 | Mark (2011) | United States | “The author provides a  general review of the concepts of evaluation use, evaluation influence and influence pathways with connections to multisite evaluations. THE study of evaluation influence and influence pathways is briefly described” (p.107). | N/A | N/A | No new findings | No new practice or  theoretical implications |  |
| 15 | Olejniczak (2007) | Poland | “In this article, we offer  serious games as a method that can be employed by evaluators to address three persisting challenges in current evaluation practice: inclusion of stakeholders, understanding of causal mechanisms, and utilization of evaluation findings. We provide a framework that distinguishes among games along two crucial aspects of evaluation inquiry – its function and the nature of the evaluand” (p. 339). | N/A | N/A | **a)** Understanding built  through serious game engagement include:  **1.** Stakeholders shared reality of the need for change. **2.** Shared understanding of the policy problem. **3.** Shared understanding of the plans for change. | **Theoretical:**  **a)** Defined serious games  as it relates to public policy and program evaluation.  **b)** Distinguished serious  games from other forms of game practices  **c)** Provided a framework  for using serious games in evaluations distinguishing between two fastest of evaluation inquiring:  **1.** function of the inquiry **2.** nature of the evaluand  **d)** Presented 4 areas of  game application in evaluation practice:  1. Teaching knowable 2. Testing retention 3. Crash-testing mechanisms 4. Exploring systems  **e)** Authors state that the  four types of games presented in this paper are useful to promote learning among stakeholders and to determine how the programs and policies operate.    **Practical:**  **a)** The authors lay out  three factors important when planning a game-based evaluation:  **1.** Clearly state the purpose of the game and the nature of the evaluand.  **2.** Evaluators need to included game designers in their planning to ensure the technology will meet their evaluation needs.  **3.** Reserve tune for calibration and testing of the gaming prototype as part of the evaluation plan. |  |
| 16 | Olejniczak (2017) | Poland | “This article explains  how to develop knowledge brokering skills through experiential learning in a risk-free environment. It reports on the application of an innovative learning method – a game-based workshop. The article introduces the conceptual framework for designing game-based learning. Then it demonstrates how this framework was applied in practice of teaching knowledge brokering.” (p. 554). | N/A | N/A | **a)** Game based learning is  emerging as experiential learning methods with features of effective learning.  **b)** Game based learning  provides a safe context to experiment, reflect in live time, seek consultations in live time all without impacting stakeholders.  **c)** Game based learning is promising for teaching evaluation skills. | **a)** “Linking the fields of  evaluation and serious gaming creates opportunities not only for teaching but also for cross-field research (p. 568).  **b)** “There is a need for  systemic evaluation studies on the effects of serious games on individuals, groups, and organizations in comparison to more traditional reaching methods” (p. 568). |  |
| 17 | Patton (2020) | United States | To respond to, reflect  on and extend Alkin and King's three article series related to evaluation use. | N/A | N/A | **a)** Acknowledge the  "comprehensive, insightful and generative" contribution of Alkin and King in their use trilogy.  **b)** Agrees with their prompt to  the reader to “pay close attention to the evolving contexts of use..." (p. 582) but he disagrees for the need of a common definition for evaluation use. Patton states that treating use as a thick sensitizing concept invites ongoing dialogue about the diverse nature of evaluation and its use. | **a)** Speaking of Coryn et al.  2017, Patton notes that seeking to identify discrete evaluation practices and definitions of use to for the purposes of replication and standardization is out of sync with the complexity theory and systems thinking.  **b)** Patton would rather  evolution research: “map interrelationships, capture diverse perspectives, document both linearities and nonlinearities, pursue both intended pathways and emergent ones, and examine the interplay between what was planned and done, what was planned and undone, what was unplanned and done, and what was omitted altogether, all in dynamic interaction, interdependence, and interrelationship” (p.599). |  |
| 18 | Patton (2007) | United States | “I shall argue that the  concept of process use is a usefulism. Safire’s playful term is what qualitative inquirers call a sensitizing concept” (p. 99). | N/A | N/A | No new findings. | **Theoretical:**  **a)** Process use should be  treated as a sensitizing concept, one that shouldn’t be measured by operationalizing and testing.  **Practice:**  **a)** Evaluators should use  process use to remind them to notice changes in their clients and make decisions about making process learning explicit in their clients. |  |
| 19 | Peterson & Skolits  (2020) | United States | “In this review, we  synthesize literature on the VfM framework and position it within a broader theory of Utilization-Focused Evaluation (UFE). We then examine mechanisms through which the VfM framework may contribute to increased evaluation use. Finally, we outline avenues for future research on VfM evaluation.” (p. 1). | N/A | N/A | **a)** Users of the framework  (and rubrics more generally) claim it is effective at improving stakeholder engagement, increasing transparency of judgments, and building trust among stakeholders, and improving use. Not a direct quote but close to the author’s original statements. | **b)** The VfM framework  offers several methods for fostering meaningful user engagement in determining program value.  **c)** Incorporating evidence  from both qualitative and quantitative data collection methods, the VfM framework may address the limitation of traditional economic evaluation in making information more relevant to primary users (p. 5). |  |
| 20 | Stugess (2015) | United States | The author’s purpose is  to share reflections on a failed evaluation. They share important contextual information about the evaluation, evaluand, client and stakeholders and reflect on poor evaluation decisions that were made. The purpose is to shed light on how evaluators ought to negotiate the terms of the evaluation and relationships to make the evaluation more useful.  “This article is  concerned with evaluation’s complicity in helping to maintain power asymmetries” (p.462). | N/A | N/A | **a)** Failure to reflect and  (re)negotiate the evaluation plan, the roles/responsibilities of evaluators, and the purposes of the evaluation lead to improper use. | **a)** This study reinforced  existing theories by using them to explain the evaluator’s experiences.    **b)** Practice Implications:  1. Disambiguate (and  negotiate) the evaluation’s purpose.  2. Clarify the evaluator’s  responsibilities and competencies.  3. Engage evaluators in  reflexivity practices.  4. Interact with the full  range of stakeholders.  5. Attune the evaluation  to cultural difference.  6. Engage developers  and funders as participants.  7. Create and protect  “safe spaces” for participants. |  |
| 21 | Svensson & Cousins (2015) | Canada | This article is a review  and integration of evaluation utilization literature with a new focus on the use of technology to increase evaluation utility.  This article focuses on  three distinct objectives:  **1.** Discuss key  theories surrounding use—mainly literature on utilization evaluation (instrumental, conceptual, symbolic) and process use—to understand how evaluators have to date viewed “usefulness” or “utility.”  **2.** Review how  constructivist thought has shifted the nature of this discussion away from identifying specific attributes such as relevance, timeliness, or evaluator credibility to instead focusing on flexibility and the importance of contextual factors. -Here, we offer our own reflections and, drawing on recent knowledge mobilization literature, propose that interactivity in communication between evaluators and key stakeholders is what may be at the root of flexibility.  3. Invite future  researchers to consider how we can increase interactivity in evaluation and discuss the extent to which technology and social media may contribute to the relevancy of our field, ensuring greater engagement of those for whom our evaluations are conducted. | N/A | N/A | **a)** Neither the evaluator nor  the stakeholder perceptions of utility are objective or permanent.  **b)** Linear communication  models seldom lead to effective knowledge use.  **c)** Knowledge building  evolved in a flexible and situation manner.    **d)** Achieving use is more likely  by ensuring the evaluation process is interactive.  **e)** Value in exploring  technology as a possible way to foster interactivity and build bridges of consensus.  **f)** Technology is meant to add  value rather than replace key interpersonal dynamics. | No Implications. |  |
| 22 | Yarbrough (2017) | United states | This article emphasizes  the foundational role of this recent scholarship and the role played by all who collaborated in planning and implementing the utility standards.  “The purpose of this  article is to describe the processes that resulted in the third edition of the program evaluation standards (Yarbrough et al., 2011) and to document how these processes and the resulting standards were informed by scholarship on evaluation use, utility, influence, and collaboration” (p.284). | N/A | N/A | a) The approaches  described in this article based on recent evaluation scholarship addressing the use and collaboration in evaluation seem to have served the third edition of the standards well. | a) Provides and overview  of the development of the utility standards, perhaps reinforcing their validity by sharing how they came to be. |  |
