# Supplementary material for: Mapping Evaluation Use: A Scoping Review of Extant Literature (2005–2022)
Source: Am J Eval. 2024 Mar 13;45(3):341–60. doi: 10.1177/10982140241234841 (PMC11469954; doi:10.1177/10982140241234841)
Supplement: sj-docx-2-aje-10.1177_10982140241234841 - Supplemental material for Mapping Evaluation Use: A Scoping Review of Extant Literature (2005–2022) [file sj-docx-2-aje-10.1177_10982140241234841.docx]

**Appendix B**

**Article Characteristics**

| Characteristics of Articles (n = 47) | Frequency (%) |
| --- | --- |
| Publication period (two-year increments) | |
| 2005–2007 | 3 (6.4%) |
| 2008–2010 | 5 (10.6%) |
| 2011–2013 | 9 (19.1%) |
| 2014–2016 | 10 (21.3%) |
| 2017–2019 | 14 (29.8%) |
| 2020–Feb. 2022 | 6 (12.8%) |
| Country |  |
| United States | 26 (55.3%) |
| Canada | 7 (14/9%) |
| Australia | 3 (6.4%) |
| Poland | 2 (4.3%) |
| Switzerland | 2 (4.3%) |
| Denmark | 1 (2.1%) |
| Germany | 1 (2.1%) |
| Netherlands | 1 (2.1%) |
| New Zealand | 1 (2.1%) |
| Sweden | 1 (2.1%) |
| Other | 2 (4.3%) |
| Article context |  |
| Evaluation discipline | 29 (61.7%) |
| Government/policy | 6 (12.8%) |
| Education | 4 (8.5%) |
| Non-profit | 4 (8.5%) |
| Health | 4 (8.5%) |
| Article type |  |
| Theoretical | 22 (46.8%) |
| Empirical | 21 (44.7%) |
| Review | 4 (8.5%) |
| Methodology |  |
| Qualitative | 9 (19.1%) |
| Quantitative | 7 (14.9%) |
| Mixed methods | 5 (10.6%) |
| Review methodology | 4 (8.5%) |
| Not applicable | 22 (46.8%) |
